# Impact of digital technologies upon teaching and learning in higher education in Latin America: an outlook on the reach, barriers, and bottlenecks

**DOI:** 10.1007/s10639-022-11214-1

**Published:** 2022-08-15

**Authors:** Kingsley Okoye, Haruna Hussein, Arturo Arrona-Palacios, Héctor Nahún Quintero, Luis Omar Peña Ortega, Angela Lopez Sanchez, Elena Arias Ortiz, Jose Escamilla, Samira Hosseini

**Affiliations:** 1 Writing Lab, Institute for Future of Education, Office of the Vice President for Research and Technology Transfer, Tecnologico de Monterrey, CP 64849 Monterrey, Nuevo Leon Mexico; 2 Division of Sleep and Circadian Disorders, Department of Medicine, Brigham and Women’s Hospital, Boston, MA USA; 3 Division of Sleep Medicine, Harvard Medical School, Boston, MA USA; 4 NOVUS Department, Institute for Future of Education, Office of the Vice President for Research and Technology Transfer, Tecnologico de Monterrey, CP 64849 Monterrey, Nuevo Leon Mexico; 5 Universidad de los Andes, 111711 Bogotá, Cundinamarca Colombia; 6 Social Sector Department, Mexico Education Division, Inter-American Development Bank, 1300 New York Avenue, N.W. Washington, D.C. 20577 USA; 7 Institute for Future of Education, Office of the Vice President for Research and Technology Transfer, Tecnologico de Monterrey, CP 64849 Monterrey, Nuevo Leon Mexico; 8 School of Engineering and Sciences, Tecnologico de Monterrey, CP 64849 Monterrey, Nuevo Leon Mexico

**Keywords:** Digital technologies, Educational innovation, Higher education, Technology-Enhanced learning, Educational technology, Learning environments, LATAM

## Abstract

Digital technology and literacy can heighten the transformation of teaching and learning in higher education institutions (HEIs). This study uncovers the extent to which *digital technologies* have been used to advance the teaching and learning process in HEIs, and the barriers and bottlenecks to why it may not have been effectively implemented across the HEIs. The study used nine selected countries in Latin America (LATAM) based on the main focus of the educators, commercial, and financial investors; to show the level of impact/implications of computer technologies on the teaching and learning processes. We applied a two-step (mixed) methodology (through a quantitative and qualitative lens) for the research investigation, using data collected from survey we administered to faculty members in HEIs across the different countries in LATAM. In turn, we implemented a Text Mining technique (sentiment and emotional valence analysis) to analyze opinions (textual data) given by the participants to help determine challenges and obstacles to using the digital technologies for teaching and learning in the region. Quantitatively, we applied a Kruskal–Wallis H-test to analyze the collected multiple choice and ranked items in the questionnaire in order to identify prominent factors that consummately influence the reach, barriers, and bottlenecks, and where the differences may lie across the different LATAM countries. The results show that the users upheld the emphasis on lack of training, infrastructures and resources, access to internet and digital platforms, as the main challenges to the teaching–learning process. The study also empirically discussed and shed light on critical factors the HEIs, particularly in LATAM, should resolve and adopt in support of the decision-making strategies, operational policies and governance, financial investments, and policymaking, at a time when “digital technologies” have become an inevitable and indispensable part of education and learning.

## Introduction

Today, modern *educational technologies* and the underlying models and practices have become an integral part of the teaching and learning process, and have showed rapid (innovative) growth within the higher education domain (Henderson et al, 2017; Mercader & Gairín, 2020; Okoye et al, 2021). As a result, many higher educational institutions (HEIs) strive to invest in digital technologies to help support the various teaching and learning processes and curriculum. Didactically, existing studies profess “digital technologies” to be one of the enabling tools that teachers, or yet HEIs, can apply to facilitate the teaching–learning processes, and improve/transform both the faculties’ and students' learning experiences and engagement (Barton & Dexter, 2020; Chiu, 2020; Livingstone & Livingstone, 2012; Sánchez-Mena et al., 2019; Tondeur et al., 2020). The digital technology have also been reported to positively impact higher education at a wider scale by increasingly providing access to learning, offering of equal learning opportunities for all, and promoting life-long learning (Juan et al., 2011; UNESCO, 2014, 2021a).

However, in this digitally-savvy age or generation of the twenty-first century; digital technologies are evolving at an unprecedented rate, although there is evidence that the pedagogical changes or transformation are slow (Boninger et al., 2019, 2020; CONECTA, 2021a; Cuban, 2020, 2021; Molnar & Boninger, 2020). In consequence, educators must consider the role and challenges that are eminent or pertinent to the use of those new and emerging technologies for learning. For example, the Technology-Enhanced Learning (TEL) notion or initiatives (Sen & Leong, 2020; Smith et al., 2021) have spanned the creation of several tools and systems that are used to facilitate the teaching–learning processes across the various HEIs. This includes, to name but a few, emergence of state-of-the-art learning tools or platforms such as: Flipped classrooms, Augmented reality (AR), Virtual reality (VR), Learning Management Systems (Moodle, Canvas, Blackboards, MOOCs), as well as, learning elements or components like Serious games and gamified learning platforms, Mobile learning (m-learning) (Er et al., 2019; Gordillo et al., 2019; Hincapie et al., 2021; Lin & Wang, 2021; López et al., 2021; Rubio-Fernández et al., 2019). Along these lines, this current study note that with support of TEL (Bälter, 2021; Chiu, 2020; Hosseini et al., 2021; Okoye et al., 2021; Sen & Leong, 2020; Smith et al., 2021), otherwise allied to the “digital technologies for education” in this paper, that learning has surpassed the need for physical infrastructure (e.g., face-to-face classrooms), and has transferred the instructional or pedagogical responsibility for Educators to provide innovative alternatives to physical infrastructures for the students (e.g., remote and distance learning, working facilities at home, technology at home) (Benabdallah & Bourgault, 2021; Chick et al., 2020; Crick et al., 2020; Jimoyiannis et al., 2020; LALA, 2020; Martens et al., 2020; Okoye et al., 2021; UNESCO, 2021b). Also, TEL-based Education (digitized-education) have attained flexibility and mobility in its mode of delivery or paradigms (Aguilera-Hermida et al., 2021; Diaz-Nunez et al., 2021; Okoye et al., 2021; del Rio-Chillcce et al., 2021; TEC, 2020b). This ranges from the innovative (pedagogical) frameworks or theories for teaching (Exter et al., 2019; Ndukwe & Daniel, 2020; Okoye et al, 2022), to the integration of educational technologies (EdTech) that are used to bridge the gap between the modern and traditional models for teaching/learning (López et al., 2021; Shambour & Abu-Hashem, 2022), and in turn, provides new paradigms or practices for achieving *sustainability* and *scalability* in the use of EdTech for teaching or educational purposes (Clark et al., 2020; López et al., 2021; Okoye et al., 2021, 2022; Tondeur et al., 2020; UNESCO, 2015, 2021b; Yu & Jo, 2014).

In Latin America (LATAM), the need for digital literacy skills and/or development of TEL-based Education have not been more than ever, emphasized both in the current literature and in practice (Cepeda-Mayorga, 2017; IEEE, 2020b; LALA, 2020; OECD, 2020a, b; UN, 2015; UNICEF, 2018; WHO, 2022). This includes, to name but a few, issues of lack of local capacity to design and build specialized educational technologies for effective learning (Cepeda-Mayorga, 2017; LALA, 2020), to limited financial resources (OECD, 2017), and inability of HEIs to leverage the information or educational datasets that are being recorded at an unprecedented rate in databases of the different institutions (e.g., lack of implementation of learning analytics) to support the administration or decision-making strategies (LALA, 2020; Mourad, 2017; Ndukwe & Daniel, 2020; Romero & Ventura, 2020; Sønderlund et al., 2019). Moreover, there is also lack of alignment between the existing educational models and the operational policies in respect to the educational labor market (LALA, 2020; OECD, 2020a, b; UNESCO, 2021a). Typically, the survey conducted by The Organisation for Economic Co-operation and Development (OECD, 2015) draws a general picture of how HEIs in LATAM are incorporating the use of digital technologies for teaching/learning, and to what extent they are adopting the e-learning programs, including their impact on the teaching and learning process in the region. The study (OECD, 2015) notes that the main challenge associated with (e-learning) distance education in LATAM, is existence of digital gap (or digital divide) (Laufer et al, 2021) in the region, as people lacking access to digital technologies or platforms are potentially excluded from the vast benefits and opportunities to TEL, that also forms one of the main ideas or issues addressed by this present study. Moreover, the quality of the offered (e-learning or digitized) programs are also adversely affected by inadequate skills and training for the teachers, and insufficient availability and access to the digital technologies and platforms for teaching and learning purposes (OECD, 2015).

Indeed, the aforenoted factors have not only been a major challenge for educators in LATAM, particularly at a time or in preparedness to the post-pandemic education era, when it has become an inevitable requirement for HEIs to ensure that the different educational services and programs for the stakeholders (e.g., teachers, students) are sustained (Bao, 2020; Kummitha, 2020; Okoye et al., 2021; Reimers et al., 2020; UNESCO, 2014, 2016, 2021a; Viner et al., 2020; Woolliscroft, 2020). There has been the stipulation as defined in the United Nations (UN) sustainable development goals (SDG) (UNICEF, 2018) that purportedly requires, including HEIs in LATAM, to adopt methods such as TEL in the modern-day learning settings. The aim must focus on acceleration of education/learning for all, irrespective of background or geographical location, through investment in world-class digital or technological solutions (UNESCO, 2014; UNICEF, 2018). Having said that, it becomes clear that modest digital literacy skills and technologies (e.g., lack of modernization or difficulty in accessing the most basic infrastructures such as internet, and ICT training/development) (IEEE, 2020b; LALA, 2020) will inadvertently result in the HEIs not being adequately prepared to participate in both the educational and labor market. Thus, leading to a limited or inadequate response to the educational needs of the region (LATAM), particularly in terms of institutional, socio-technical, communal, productivity, or commerce. The above limitations would only heighten the disparaging demographic and social conditions that are being faced in the region. Henceforth, teachers and students who tend to be the direct consumers of the infrastructures/technological provisions need to be provided with hands-on practical, valuable, real-life work-related digital skills and literacy, to compete in the modern-day educational and labor market at large (UNESCO, 2014).

### The rationale of the study

Although the modern-day educational technologies have shown to be effective and promising towards teaching and learning (Ferguson et al., 2014; Herodotou et al., 2019; Ndukwe & Daniel, 2020; Sánchez-Mena et al., 2019). TEL-based education requires new skills (digital literacy) including institutional infrastructures to support the transition from traditional learning spaces/environments to the web-based (digital) learning platforms (Okoye et al, 2022; Shambour & Abu-Hashem, 2022). Notwithstanding the different educational initiatives (either deployed or ongoing), this study notes for the past eight years, only one comprehensive study (UNESCO, 2012) has been conducted to assess the prominence and application of digital technologies in education in LATAM. Therefore, this current study offers a robust incentive or empirical approach to understand and generate insights into the status quo, barriers, and bottlenecks pertaining to the application of digital technologies and as a tool towards attaining a more effective TEL-based or Digitized-Education and Innovation in LATAM. We believe that the awareness or discernments that this study provides does not only help to improve the teaching and learning processes across HEIs in LATAM. But also, helps to support and put forward the magnitudes or practices that educators and policymakers, both in LATAM and internationally, should absorb in their various educational settings and governance at large.

The research questions of this study are as follows:

1. What are the reach, barriers, and bottlenecks to the use of digital technologies upon teaching and learning process in HEIs in LATAM?
2. How does the identified reach, barriers and bottlenecks differ across the LATAM countries?
3. How can the findings be used to support the pedagogical practices, decision-making, and governance in HEIs in LATAM?

Based on the stated research questions and objectives, this study makes the following contributions to knowledge:

1. It provides an empirical study of prevailing factors that impacts the use of digital technologies upon facilitating the teaching and learning processes across HEIs in Latin America (LATAM).
2. It determines the reach and barriers to use of digital technologies for teaching and learning in HEIs in the LATAM region.
3. It uncovers potential bottlenecks on why digital technologies may not be effectively implemented in the higher educational institutions.
4. It demonstrates the benefits of data-structure approach such as the Text mining technique and its application within the educational domain or context, to understand the impact of digital technologies for teaching and learning.
5. It provides information on the state-of-the-art and implications of using the digital technologies to support the different pedagogical practices, decision-making strategies, and operational policies or regulations for the educators.

## Background information

### Digital technologies towards educational innovation: global perspective

In the modern education settings of the twenty-first century, increase in the use and application of digital technologies has made education a global asset (Bezanilla et al., 2019; Klein et al., 2019; LALA, 2020; Pedró et al., 2019; Romero & Ventura, 2020; UNESCO, 2014). Globally, many educational institutions strive to establish and implement TEL-based initiatives for their several didactic activities with the aim to help create an easy and interactive learning environment (UNESCO, 2014, 2021a, b). The goal is not only to create new (techno-based) learning environments that are engaging and intuitive for the learners, but are also amenable to accommodate the digitally-savvy generation (UNESCO, 2014). As an example, in recent time, UNESCO has made global citizenship education (GCE) (UNESCO, 2014) one of its central educational objectives for the period of 2014 to 2021 (UNESCO, 2014, 2020, 2021a). This was in response to the increasingly demand for its Member States to participate in empowering learners to become conscientious global citizens, particularly during and in lieu of the recent and unprecedented time of the global pandemic (Covid-19) that have disrupted the teaching and learning processes in HEIs (OECD, 2020b; Okoye et al., 2021; Reimers et al., 2020; UNESCO, 2020, 2021a). In consequence, governments and policymakers have been making tremendous efforts to support and accommodate ICT in education (TEL-based Education) by leveraging and embracing its usage for teaching and learning (IDB, 2020; Kummitha, 2020; Pagés et al., 2020; UNESCO, 2014, 2020, 2021a).

The use of TEL in education is already a global phenomenon (Bälter, 2021; Sen & Leong, 2020; Widger et al., 2016). Its implementation and status quo vary due to different factors, including economic situations of each national settings, e.g., cross-cultural or cross-national pedagogies, when one takes into account the technological gap between the developed and developing nations (Aguilera-Hermida et al., 2021; Bezanilla et al., 2019; LALA, 2020; Sánchez-Cruz et al., 2021; UNESCO, 2014, 2021b; Widger et al., 2016).

### Factors promoting the use of digital technologies in education

#### Digital skills and literacy

Nowadays, modern education (learning) frameworks requires the students and faculties to acquire or possess multi-skills, including digital literacy required for work and citizenship, self-education, life-long learning and acquittance (Barton & Dexter, 2020; Dede, 2010; Lin & Wang, 2021; Ma et al., 2021; OECD, 2021; Okoye et al., 2021; UNESCO, 2014, 2021b; Urbancikova et al., 2017). Those multi-skills which include creativity, problem-solving skills, critical thinking and analysis, among others, enable students to learn and attain sophisticated (learning) competencies that are necessary for prosperity, and effective time and content management (Okoye et al., 2021; Seyfried & Reith, 2019; UNESCO, 2015, 2020b). Moreover, the stated competencies are facilitated in a bid to allow the students to compete in a vying education environment and market in which they are held to have a competitive edge (UN, 2021; UNESCO, 2014, 2015, 2016). Consequently, many countries strategize different initiatives for investing on digital technologies which, all in turn, are aimed to support and develop the stated competencies, or yet digital learning skills for the stakeholders per se (CONECTA, 2021a; Garcez et al., 2021; INEE, 2019; Martens et al., 2020; Mikheev et al., 2021; Munro, 2018; Toit & Verhoef, 2018; UNESCO, 2021b; Urbancikova et al., 2017).

#### Digital technology and infrastructural investment

The integration of digital technologies in education requires great investment coupled with capital and human resources (CONECTA, 2021a, b; Haruna et al., 2019). Many countries have failed to afford not only the resources that are needed for the so-called TEL-based education (digitized-education), but also in consequence, have failed to integrate fully, digital technologies in the different educational ecosystem or contexts. While many developed countries have invested in digital technologies, many developing nations face an arduous and ominous task of doing so, primarily due to the inherent costs (Tsegay, 2016; IEEE, 2020b; Sánchez-Cruz et al., 2021). In Europe, for example, many countries have set aside large amounts of funds and resources for investing in and supporting the attainment/integration of digital technologies in education (European Commission, 2018). In particular, the European Commission (2018) has been supporting digital technologies in education, policy, and initiatives by funding research and innovations aimed to foster the scaling up of the teaching and learning processes. Noteworthy, under the Horizon 2020 Framework, Seventh Framework Programmes for Research and Technical Development (FP7), and Competitiveness and Innovation Framework Programme (CIP); the European Commission budgeted 80 billion Euros to support the conducting of research and fostering of innovation in a digital-aided manner from 2014 to 2020. Previously, the European Commission have also invested a significant amount of 183 million Euros into supporting research and innovation in TEL from 2007 to 2013 (European Commission, 2018).

Prior studies have also reported that for the past few years, up until 2014, the United States have instilled and spent more than $3 trillion (USD) to facilitate digital technologies for education, spending around $809.6 billion per year towards the availability and use of digital technologies in classrooms (Fredrickson et al, 2014). Those type of investment, perhaps, may have contributed to a significant transformation of the educational system in the US, in comparison to the other countries or regions, by ensuring that digital technologies are effectively implemented in education, especially during the recent pandemic (Aguilera-Hermida et al., 2021). Nevertheless, although, the existing studies indicated that a well-funded campaign to promote virtual education has succeeded in broadening the scope and expanding the reach of digital/educational technologies across the US, and the recent global pandemic has created a new need for virtual technologies, particularly in education (Boninger et al., 2019, 2020; Molnar & Boninger, 2020). It is noteworthy to mention that the fact remained that little to no research have been done to uncover the educational efficacy. For instance, studies have questioned the transformative practices of the digital technologies at different levels of education in practice (Cuban, 2020, 2021; Molnar & Boninger, 2020). Ranging from assessment and classroom teaching and learning, to commercial implications of the virtual education (Cuban, 2020, 2021; Molnar & Boninger, 2020), which portentously can form an important future areas of research, both in theory and in practice, particularly as it concerns unveling the educational efficacy, interconnectedness, or deficiencies between TEL-based education and the educators’ funding campaigns or instructional infrastructures.

#### Digital technologies transformation and evolution

With “transformative education” being at the center of many HEIs goals (Cantón, 2018; Takayanagui, 2017; Winthrop & Barton, 2018), the National Educational Evaluation Policy Gazette in Mexico (INEE), for instance, noted that “digital technology” has spurred particularly the educators to consume, innovate, and transfer knowledge/practices that transcends the teachers and students into becoming global voices (INEE, 2019). One of the most occurring problems in the region (LATAM) is the issue of digital transformation, especially during the pandemic, hand-in-hand with digitized-teaching and scalable strategies for the development of the teaching–learning process. However, it is important to mention that Educators are not relenting in ensuring that the congruence of the “technology” and “education” is well promoted, embraced, and improved across the regions. For example, the Tecnologico de Monterrey (TEC) and Universidad Nacional Autonoma de Mexico (UNAM) have recently signed a partnership agreement to promote the Scientific ecosystem and Technology of the host country (CONECTA, 2021b). The consortium which amounts to a total investment of $20 million Mexican Pesos was aimed to reinforce technological projects, synergize with companies, and endorse a commitment to Science, Technology, and Innovation (STI) for the development of the nation (CONECTA, 2021b). Besides, the authors believe that massive investment in digital technologies is necessary to enhance the teaching and learning processes and the several associated activities (CONECTA, 2021b; UN, 2021; UNESCO, 2021a). The level of digital technology literacy for both the instructors and learners can help heighten the transformation of teaching and learning processes or practices in education (OECD, 2016). Thus, the level and/or impact of digital technology literacy is much conditioned by the availability of digital tools, and vice versa. For example, many countries that are considered “high-tech” by virtue of their technological development and/or extent of their application, have citizens with higher digital technology literacy levels than their counterparts in low-tech settings (Ding, 2020; Haruna et al., 2019; Pan & Fan, 2020). As such, countries that invest in digital technologies tend to use them in diverse socio-economic or life spheres, including for teaching and learning purposes, as their acquired or manifested skills can critically promote educational innovations (OECD, 2016). To support this important development, the United Nations Educational, Scientific and Cultural Organization (UNESCO) has conducted a seminar aimed to capacitate educators from Asian and European countries in order to equip them with relevant knowledge and skills to apply digital technologies (e.g., web-based or online learning, massively open online courses (MOOCs)) for improving learning outcomes in HEIs (UNESCO, 2018), and to note, in preparedness to the recent time of the pandemic or post-pandemic education (UNESCO, 2021a, 2021b).

#### Digital technologies for learning in urban–rural context

Although high-tech countries have made a massive investment in digital technologies in education and its practices, their counterparts in low-tech settings still struggle to transform the underlying education systems to cope with the (state-of-the-art) globalisation and digitally-savvy generation’s learning and life-style (Lakkala & Ilomäki, 2015; UNESCO, 2015). This includes the critical or yet ample need for adequate TEL-based learning, due to poor resources at their (low-tech) disposal. On the other hand, notwithstanding that high-tech nations have invested immensely in digital technologies, their applications in real-time encounters various challenges due to the narrow impact of digital technologies in transformation of the educational ecosystem at large (CONECTA, 2021a; OECD, 2015, 2016, 2021). Despite the OECD’s Innovation Strategy and emphasis on investing in infrastructures such as hardware and software; the potentiality of improving the stakeholders’ (e.g., teachers and learners) digital literacy skills, enhancing educators’ professional development, reforming instructional methods, production of customized software and courseware, among many others, are yet to get full consideration (OECD, 2016).

Apart from having different initiatives and strategies for educational transformation or process, the counterparts in low-tech settings, have faced challenges including the shortage of funds for investing in digital technologies, low digital literacy skills by the stakeholders, and limited expertise in the use of digital technologies for education (Haruna et al., 2019). Furthermore, Haruna et al. (2019) notes that in low-tech settings, it seems that the stakeholders are not ready (or sometimes reluctant) to initiate the much-needed transformation to TEL due to the lack of awareness, limited infrastructures, such as connectivity and network, and limited internet bandwidth (IEEE, 2020b; Mercader & Gairín, 2020; UNESCO, 2015).

However, in some cases, the digital infrastructures and the linked products/devices are not designed to be operated in low-tech settings. As a result, the low-tech settings per se, are forced to improvise in the use of ready-made products offered by the developed nations which may be used in high-tech settings with repercussions (Lakkala & Ilomäki, 2015; UNESCO, 2015). Additionally, due to dire economic straits prevailing in the low-tech settings, some countries may fail to afford the costs related to procurement and deployment of digital devices such as laptops, computers, mobile phones, tablets, and internet subscriptions for teaching and learning purposes (Parliamentary Office of Science & Technology, 2006; Swarts & Wachira, 2010). Henceforth, addressing the digital divide or gap factors (Laufer et al., 2021) is critical to fully transform the largely wobbling education systems, into a digitally-aided and enabled one that supports the teaching–learning processes.

### The state-of-the-art and current situation in LATAM

#### Educational policies and action plans

Like any other nation in the world, countries across the LATAM are maximizing their efforts in implementation of global initiatives for transforming their various educational systems, especially to alleviate the current learning needs (IDB, 2020; OECD, 2020b; Pagés et al., 2020). Perhaps, those initiatives have spanned in order to make the graduates competitive in the global economy (UNESCO, 2014). For instance, through the acquisition of relevant/new sets of competencies and digital literacy skills. Indeed, LATAM countries showed a commitment to fastening the transformation of digital technologies in its education ecosystem or yet TEL, by developing policy-based frameworks and action plans. For example, in 2011, a regional call upon educational institutions in the region took charge of integrating digital technologies in their respective education systems (ECOSOC, 2011). Also, in 2013, an action plan was developed that prioritized the integration of digital technologies by focusing on fostering world-wide access and inclusive education (ECLAC, 2013). In those veins, member states reached a consensus on four goals for education reinforced by the Lima Declaration (ECLAC, 2013, p. 11):

i. Goal 23: Support collaborative teaching and research activities by ensuring high network and infrastructural access, and promoting the use of convergent educational resources such as mobile phones, video games, and open interactive digital television and platforms, etc. Thus, strengthening the regional network for Science, Technology, and Innovation (STI).
ii. Goal 24: Ensure that all stakeholders (teachers, staff, and management) in the HEIs have basic ICT training and skills, that enables them to incorporate the technologies effectively into their various teaching–learning process.
iii. Goal 25: Encourage the development of interactive educational applications, and promote the production of open educational resources (OER) based on the principles of accessibility, user-friendliness, and free-availability.
iv. Goal 26: Promote cultural diversity, e.g., through The Latin American Network of Educational Portals (RELPE) in exchange, joint production, and generation of shared repositories of multimedia contents, distance training, and teaching models*.*

Likewise, between 2010 and 2011, UNESCO conducted a survey to evaluate the integration of digital technologies in the education systems in LATAM (UNESCO, 2012). The results showed that many countries (82%) developed formal written documents that stipulates the transformation to digital-inclined education and initiatives. Moreover, some countries indicated having digital technology literacy skills as inclusive in the educational curricula (UNESCO, 2012).

#### Digital technologies for educational research and development

In LATAM, several projects have been developed that focus on embracing the role of digital technologies in sustaining the teaching and learning processes (i.e., technology-mediated education or TEL), and elimination of the bias or preconceptions of adopting new educational technologies (EdTech) for learning. As an example, the Students4Change project was a program developed and implemented in LATAM which was supported with funds from the European Union within its Erasmus + Capacity Building in the field of Higher Education program (Cepeda-Mayorga, 2017). The program (Student4Change) was aimed at development of competences in entrepreneurship and social innovation as part of the curriculum plan in HEIs in LATAM, that allows students to identify and propose solutions to the social problems that afflict the region. Also, a separate study carried out by Mexico’s Autonomous University (UNAM, 2020), one of the largest public universities in the LATAM region, in context of moving classes online as a result of the recent Covid-19 pandemic, has shown that the two most prevalent challenge or difficulties amongst teachers and students are specifically (i) poor knowledge of pedagogical possibilities of TEL-based Education (pedagogical challenges), and (ii) uneven access to technology or adequate infrastructures (socio-technical or infrastructural challenges).

Moreover, The Learning Analytics Community in Latin America (LALA) records that there is a lack of local capacity to design and build specialized educational technologies that can be utilized to improve the teaching–learning processes for the different HEIs in LATAM, in contrast to its European counterparts (LALA, 2020). The LALA project (LALA, 2020) is an international open access initiative formed by HEIs and related companies in LATAM to help “improve operations and management of the HEIs” and provision of “quality assurance mechanisms and learning processes”. The aim of the project (LALA) is directed towards modernization and improvement of academic decision-making strategies and processes at different levels within the HEIs, including the quality, efficiency, and relevance of higher education in LATAM, respectively. It functions under the priorities set for LATAM inside the Erasmus + project requirement for building educational technologies or local capacity to design and implement Learning Analytical (LA) tools (Ferguson, 2012; Ferguson & Clow, 2016; Papamitsiou & Economides, 2019; Romero & Ventura, 2020) to facilitate education across the HEIs in LATAM. In practice, the LALA framework (LALA, 2020) depicts institutional, communal, methodological, technical, and ethical aspects to deploying learning analytics (LA) or distance learning within the context of HEIs in LATAM. For example, the project (LALA, 2020) highlights that most academic decisions by the HEIs especially in the last decade of modernization of academic system in LATAM, are based on feelings or preconceptions. They further argued that if the large amounts of educational datasets, that are being generated every day and usually being ignored by the HEIs, are effective utilized, that not only will the most basic levels of information be made available for the users, but also the resultant tools and results of its use and analysis can be used to ensure a valuable impact on operations of the HEIs in LATAM at scale. Along the same lines, this current study showed and strongly believes that adequate adoption and implementation of educational technologies or methods, such as the *learning analytics* (Ferguson, 2012; Papamitsiou & Economides, 2014, 2019; Romero & Ventura, 2020) or yet *teaching analytics* (Ndukwe & Daniel, 2020; Wise & Jung, 2019), will do no harm but instead beneficial for the different HEIs in LATAM. Ranging from developing and provision of digital platforms for the instructors to communicate effectively with the students, to the extraction and storage of the active users' data (IEEE, 2020a) for further learning process analytics, management, monitoring, and recommendations. Moreover, the results of the resultant methods per se, can be utilized to support the overall goal of the HEIs and learning strategies, including re-design of the administrative systems, if needed, to suit the different learning needs of the stakeholders. In turn, enabling or ensuring a flexible and effective learning management systems (LMS) for the HEIs, facilitation of hands-on digital and online practical sessions for the students and faculties through adequate digital literacy skills and technologies, and maintenance of all service standards at the highest achievable levels, with emphasis on education quality, students care, and sustenance of learning, especially amidst and in preparedness to the recent global pandemic (COVID-19) post-education era, including infrastructural development and cost-effectiveness or use of all available resources.

#### Digital ecosystem and pedagogy in LATAM

Recent study carried out by the Organization for Economic Co-operation and Development (OECD, 2020a) has delved into the complex system of the LATAM countries, as example, the Mexican education system, and has identified one important challenge in its current iteration; a lack of alignment between the current policies and educational models with the labor market. While there is continuous effort to expand the presence and support of education programs throughout Mexico, recent events such as the Covid-19 pandemic have accentuated a lack of formal training and infrastructure to continue the education of young adults. This problem is further unveiled in those communities who have difficulties accessing even the most basic required learning materials or infrastructures such as internet or other TEL-based tools (Sánchez-Cruz et al., 2021). According to the OECD’s report (2020a), between 2007 and 2017 attaining higher education among 25 to 34 year-olds in Mexico rose from 16 to 23%, although this is still far below the OECDs’ average (44%), and below countries such as Colombia and Chile (30%). There is also a huge disparity along the ethnic or cultural lines. In 2015, only 6.6% of indigenous 25- to -64 year-olds (OECD, 2020a) had completed a higher education degree, compared to almost 19% in the rest of the population in Mexico. Although, in the academic year of 2017/2018, there were 4.5 million students enrolled in higher education in Mexico, with 2.4 million more than figures recorded in 2000. The outcome of this study shows that those challenges or shortcomings, such as lack of alignment of the educational models with the labor market and policies, will continue to impact the level of success in education in LATAM. Unless the educationalists, policy makers, and investors, in LATAM, take into account the levels of digital literacy skills, availability of resources or funds, and technological infrastructures for the stakeholders (e.g., HEIs, teachers, students), aimed towards attaining and ensuring a continuous and effective learning experiences or education for them. Some of the outcomes of this study putatively aligns with the aforementioned factors which we empirically discussed in detail in the Discussion section (Section 4).

Recently, a qualitative study was conducted in Brazil and Mexico to assess the impact of educational technologies in education (Jassir, 2018). From the holistic point of view, the study projected that the number of internet users in 2021 would reach 61% in Brazil and 50% in Mexico. The study (Jassir, 2018) indicated that there is a considerable number of information and communication technology users in both countries, and the number is sufficient for establishing digital technologies in education. Interestingly, the results of this study (see Sections 3.3.1 and 3.3.2) also shows that the lack or limitation of digital literacy skills and training is a compounding factor on the use of digital technologies for teaching and learning in HEIs in LATAM. Furthermore, there is also the stipulation that transformation of educational technologies started a decade ago when the massive open online courses (MOOCs) and learning management system (LMS) was introduced (Jassir, 2018). Thereafter, the use of digital technologies and innovation in education consequently increased. Moreover, according to Jassir (2018), 15,000 EdTech companies applied for the educational start-up capital competition in 2018 aimed to design various digital technologies to help improve teaching and learning (e.g., courseware and curricula). It is also worth noting that modern educational institutions, such as Tecnologico de Monterrey, have conducted a study which reports that there are nearly 150 to 180 digital technology for education companies in the LATAM region (TEC, 2020a). Moreover, the private institution in LATAM (TEC, 2020a) has developed under its Institute for Future of Education (IFE) initiative, a digital for tertiary education program (D4TEP) aimed to support digital transformation of HEIs in LATAM, and third world countries in the Caribbean region and continent of Asia (IFE, 2020). Upheld from the findings of this study, the goal is to develop educational programs that helps accelerate plans for the HEIs, towards developing and improvement of their digital transformation strategies, and then as a roadmap for the future of education and sustainability in the diaspora. Also noteworthy, is the Flexible Digital plus Model (MFD +) developed by the HEI (TEC, 2020b) which includes different technologies to support academic continuity (e.g., Canvas, Blackboard), Life at home program, Emotional health of students and their families, and Boost your skills programs (designed to complement learning for the students using platforms such as edX and Coursera, via MOOCs and webinars) (Okoye et al, 2021). Utterly, the HEI has developed the stated didactic elements and model in order to ensure that learning and training experiences of the stakeholders are maximized, even under unusual circumstances such as the recent pandemic (TEC, 2020b). Thanks to trends and advancements in digital technologies and literacy that have formed not just an integral part of the modern educational models by the higher institutions. But also, have become an effective tool in development of disciplinary and transversal competencies that include Challenge-based learning, Memorable university experience, Inspiring professors, and Flexibility as to how, when, and where learning occurs, to achieve the goals of the educational institutions (TEC, 2018).

#### Digital technologies and infrastructure investment in LATAM

To improve education across the region, LATAM has spent more than $2 billion (USD) since 2008 up to 2019 to reform the education systems, which are deployed as digital technology innovation initiatives and are aimed to improve teaching and learning (IDRC, 2019). The investment in the digital education initiatives, however, seems not to be effective as anticipated, since the intended outcome of improving the teaching and learning processes has not been met (IDRC, 2019). The authors note that such ineffectiveness could be associated with the identified challenges, including the unavailability of digital technology or devices due to factors such as severely curtailed digital literacy skills, training, and funds. Estimate by the International Development Research Center (IDRC) notes that to address this situation, the IDRC awarded $1.3 million (CAD) towards its innovative projects estimated to last for a period of three years in total. While the Fundación Ceibal and ANII; the National Research and Innovation Agency of Uruguay (Agencia Nacional de Investigación e Innovación) also capitalized $1.5 million (USD) to support the same ongoing projects and initiatives, purportedly aimed to improve digital education in LATAM (IDRC, 2019).

Other challenges stipulated are educators not being involved in the different initiatives, as instructional practices are not integrated in the different projects or curriculum, and there is also lack of accountability and discrepancies in monitoring and evaluation of the approaches (implemented or ongoing) or transformational mechanisms (UNESCO, 2012).

## Methodology

A two-step (mixed) methodology was applied in this study (Creswell & Creswell, 2018; Seyfried & Reith, 2019). Whereas the study have used both quantitative and qualitative datasets derived from the survey we conducted to understand the reach, barriers, and bottlenecks to use of digital technologies in LATAM. It is also important to mention that our research approach (through the quantitative and qualitative lens) was grounded on the descriptive and diagnostic research design (Narayan, 2019), that focuses not only on describing the situation or constructs we have studied in this paper based on the stated research objectives, but also it allowed us to examine/explore the potential and/or underlying causes of the identified barriers or bottlenecks we have found (Twining et al., 2017). Thus, through the use of the mix of method, the research theoretically studied the opinion or perspective of the faculties as it concerns the use of digital technologies in education, collected through the survey questionnaire that consists of both the quantitative (numeric) and qualitative (non-numeric) items, within the positivism framework or theory that upholds research knowledge/investigation from the experience of natural phenomena and their connected properties or relations by describing those in an analytical and tautological manner (Elden, 2009; Outhwaite, 2015; Turner, 2001).

For the data collection, an online survey was created and administered using the Qualtrics.XM survey platform (Qualtrics, 2020) (see: Appendix 1). The survey questionnaire was applied in the first quarter of 2020 (pre-Covid) and analyzed during the Covid-19 pandemic. The distribution of the survey was done through a paid social media campaign. Also, the survey was posted on the Observatorio de Innovacion Educativa platform (Observatorio, 2020), the newsletter of the Institute for Future of Education (IFE, 2020), Center for Educational Innovation of the host Institution (TEC, 2020a). The Observatorio newsletter specializes in all topics related to educational innovation and provide various education-related products at international scale, to disseminate knowledge and create awareness that drives research, innovation, and entrepreneurship in educational innovation, by collaborating with professionals around the world. The initiative is aimed to meet today's educational challenges and craft the future of education.

Targeted participants were selected from nine purposively selected countries to represent the main regions in LATAM (Argentina, Chile, Brazil, Mexico, Peru, Costa Rica, Ecuador, and Uruguay) by taking into account the primary focus of the educational, commercial, and financial investors (IDB, 2019; LASPAU, 2020; Microsoft, 2020; TEC, 2020a) that covers the large margin of the region’s population with major economies and top HEIs that are part of the Ibero-American association for distance higher education (AIESAD, 2020) within the emerging fields of TEL (Brunner & Ferrada, 2011; OECD, 2015). The collected data was primarily from faculty members from the several higher educations and learning institutions across the selected countries in LATAM.

### Research instrument

For the survey instrument, three main factors or constructs were considered whilst designing and administering the questionnaire. The study looked at the impact of digital technologies for teaching and learning process in LATAM by considering, (i) the demographic information and reach, (ii) the extent or barriers in use of digital technologies for teaching and learning, and (iii) the bottlenecks on why digital technologies may not be effectively implemented in the higher institutions. It is important to mention that by “reach” the authors refer to demographic distribution or extent to which digital technologies have been used to harness teaching and learning processes in HEIs in LATAM. Whereas, “barriers” refers to both the external and internal obstacles such as limited resources, lack of technical capabilities and support, or social status quo that could affect the integration of digital technologies for learning in the different HEI settings. By “bottleneck” we refer to the several factors in terms of using or adopting the digital technologies for teaching and learning that could cause the process to slow down or affects the practical application and/or performance of those technologies (Raza, 2019; van der Aalst, 2016).

The survey instrument went through several stages of validation in order to ensure the reliability and validity of the collected data. Several pilot tests were applied to ensure the relevance and validity of the questionnaire before sending it to the participants. This includes initial administering of the questionnaire to a few number of faculties to get their feedback on the survey, and several focus group discussions was also carried out by a group of experts within the educational innovation research domain, to have a clear understanding of the connotations and evaluation mechanisms of the constructs that we considered in this study (i.e., the reach, barriers, and bottlenecks in the use of digital technology for teaching and learning in HEIs in LATAM) (Brown, 2019).

The estimated minimum sample size for the research was 40 participants, which we considered to be a scientifically acceptable large enough sample size (*n* > 30 or 40) (Roscoe, 1975) for conducting the statistical analysis and procedures in this study. The application and completion of the survey was voluntary, and it took approximately 20 min to be completed. The survey was provided and administered in three different languages (English, Latin-American Spanish, and Portuguese) to cover the targeted countries, with the back-translations checked by professional native speakers to ensure the conformability and validity of the survey items considering the various linguistic and cultural provinces in LATAM. Henceforth, the main factors (i.e., reach, barrier, and bottlenecks) underlay the different items and participants’ responses to the survey questions. The participants answered a 37-item questionnaire with both ranked Likert-scale, multiple choice, and open-ended questions. Given that the questionnaire Items were a combination of ranked Likert scale and multiple-choice questions with varying scales of measurement, including also an open-ended question, the study applied a factorial analysis (Cortina, 1993; Green et al., 1977; Jasper, 2010; Tate, 2003; Tavakol & Dennick, 2011) for the purpose of comparison and validation of the dataset, sample size, and results of the analysis (see: Table 1). The Principal Components factor Analysis (PCA) with Varimax Rotation (Allen, 2017; Brown, 2019) was used to analyze the survey items to determine its reliability and adequacy in measuring the research constructs and objectives. The results of the PCA analysis shows that the survey items where valid and reliable (adequate) for testing the research constructs; with Kaiser–Meyer–Olkin Measure of Sampling Adequacy (KMO) = 0.690, Bartlett's Test of Sphericity = 7748.76, and significant value *p* = 0.000, where Eigenvalue > 1, (Ermatita et al., 2019; Frost, 2021; Goni et al., 2020; Sevincer et al., 2017). It is important to mention that the last question in the survey (Item 37) was a text-based open comment question, “In your opinion, what are the obstacles or challenges to using digital technologies for teaching–learning in higher education in LATAM?”, that was asked to the participants, and therefore, was not included in the PCA test. Also, when we further analyzed the individual items in the study questionnaire that primarily targeted (construes) the three different constructs (i.e., reach, barrier, and bottleneck) (Table 1) we have developed based on the theoretical framework described in the methodology, using the PCA factor analysis; we found that the items were valid and adequate for measuring the stated constructs, as reported in Table 1.

**Table 1:** Principal Components Analysis (PCA) with Varimax Rotation factor analysis for the three Constructs (Reach, Barrier, Bottleneck) and survey items

	Principal Components Analysis (PCA) with Varimax Rotation, Eigenvalue > 1				
Construct (Factor)	Item (question)	Scale	KMO	Bartlett’s Test	*p*-value (Sig.)
Reach	18,19,24,17,25,26	Ranked Likert, Multiple-choice	0.520	106.65	0.000*
Barrier	16,20,21,22,23	Ranked Likert, Multiple-choice	0.514	1081.56	0.000*
Bottleneck	27,28,29,30,31,32,33,34,35,36	Ranked Likert, Multiple-choice	0.704	3994.27	0.000*

Significance level: *p* ≤ 0.05, Items (question) description is provided in Table 5

In summary, the survey instrument based on the aforementioned framework and analysis was designed and administered by considering the following three factors (constructs):

- *Factor 1* covered demographic information and extent of reach.
- *Factor 2* covered the barriers in the use of digital technologies for teaching and learning.
- *Factor 3* covered the bottlenecks on why digital technologies may not be implemented in higher education in LATAM.

### Data sampling

**Fig. 1:**
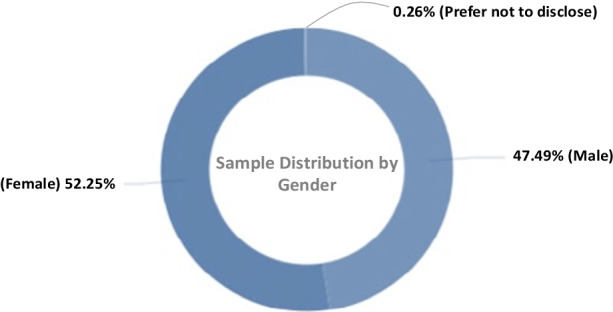
Demographic distribution of participants based on the gender

**Fig. 2:**
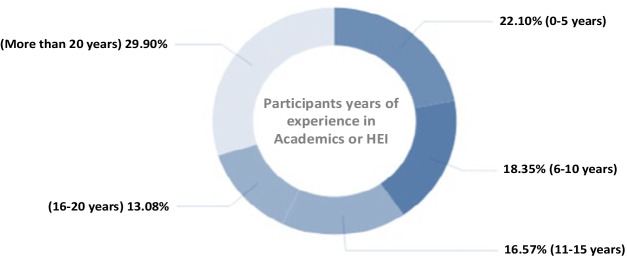
Distribution of participants based on number of years or experience within the HEI

**Fig. 3:**
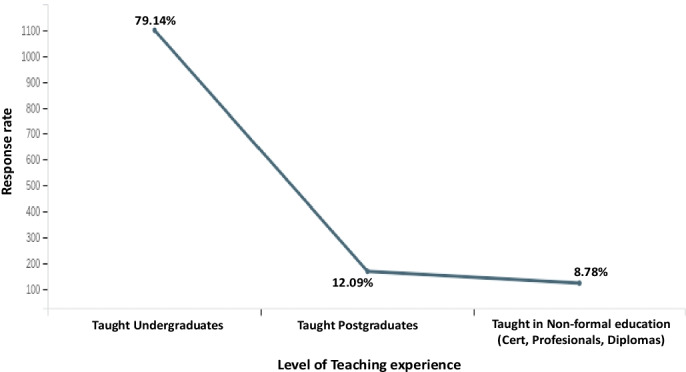
Distribution of participants based on their Level of Teaching experience

**Fig. 4:**
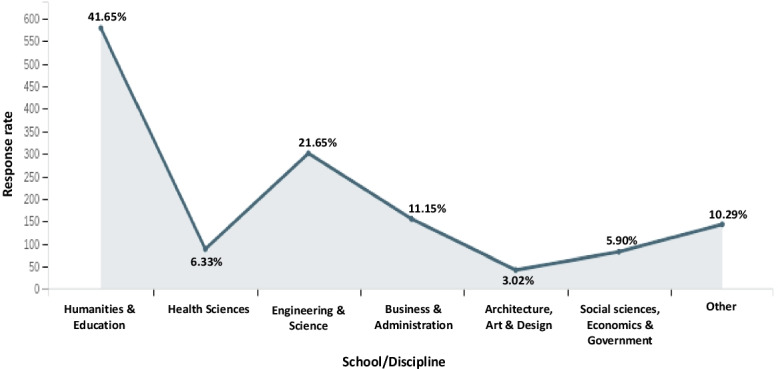
Distribution of participants based on the School/Discipline

**Fig. 5:**
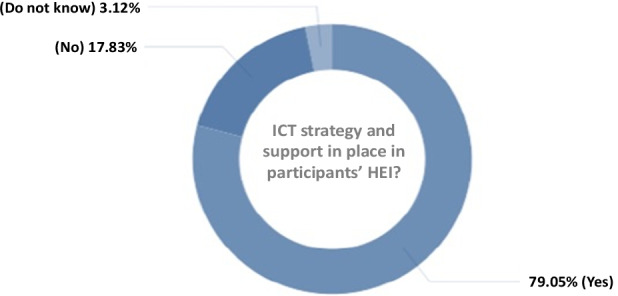
Distribution of participants in terms of whether ICT strategies and support are in place in the HEI

The response rate for the administered questionnaire was a total number of *n* = 1576 participants who responded to the survey invitation across the different LATAM countries. Considering the demographic information and descriptive statistics of the collected dataset as shown in Tables 2, 3, 4, and Figs. 1, 2, 3, 4 and 5); the study notes that 47.49% of the respondents were males, 52.25% females, and 0.26% did not prefer to disclose their gender (Fig. 1).

**Table 2:** Level of academic qualifications or degrees of the participants

Academic qualifications/degrees of the participants	
Level	Percentage (%)
Bachelors	26.29%
Masters	47.43%
Doctorate	16.83%
Other	9.46%

**Table 3:** Table showing whether there are strategies for incorporation of digital technology in delivery of the Courses in the HEI

Integration of digital technologies in delivery of Courses	
Level	Percentage (%)
Yes	56.76%
No	11.22%
Special occasions	30.79%
No applicable	1.23%

**Table 4:** Cost of licensing of digital technologies or software for teaching in the HEI

Cost of licensing of digital technologies and software in delivery of the courses	
Level	Percentage (%)
High barrier	42.40%
Enough barrier	30.81%
Little barrier	22.28%
Not a barrier	4.51%

The level of academic qualifications or degrees of the participants or faculties was; Doctorate (16.83%), Masters (47.43%), Bachelors’ degree (26.29%), and Other (9.46%) (Table 2). Of which 29.90% agreed to have more than 20yrs of experience in the HEI settings, 13.08% between 16-20yrs, 16.57% 11-15yrs, 18.35% 6-10yrs, and 22.10% 5 years and below, respectively (Fig. 2).

79.14% of the participants have taught or teaches the Undergraduates (Bachelors’ degree), 12.09% taught the Postgraduates, and 8.78% for Non-formal education (i.e., certifications, profesionals, diplomas, etc.) (Fig. 3).

The main disciplines in which the participants or faculties have taught include: Humanities and Education (41.65%), Health Sciences (6.33%), Engineering and Science (21.65%), Business and Administration (11.15%), Architecture, Art and Design (3.02%), Social sciences, Economics and Government (5.90%), and Other (10.29%) (Fig. 4), with 94.68% of the participants from Formal Educational institutions, while 5.32% came from Non-formal settings. The type of Institution in which they are affiliated included Public (53.74%) and Private (46.26%).

79.05% of the respondents indicated that their educational institutions have an Information and Communication Technology strategies or support in place, while a total of 20.95% do not have (17.83%) and do not know (3.12%) (Fig. 5). Of which 75.04% agreed to use or have used technological platforms for learning (such as Canvas, Blackboard, Google classroom, etc.), and 19.75% do not, or do not know (5.21%). Also, 90.35% of the faculties (totally agree = 58.97%, agree = 31.38%) claimed to have used digital technologies such as specialized software, simulations, video production, virtual reality, etc., for teaching purposes and/or delivery of their courses, while 9.65% disagreed (7.56%) or totally disagreed (2.09%).

**Fig. 6:**
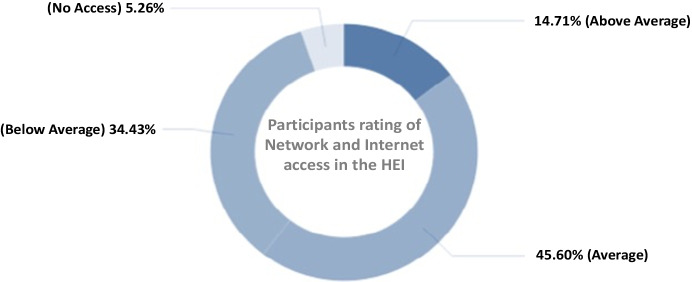
Distribution of participants’ response based on availability of Network and Internet access across the HEI

In terms of Internet access and Course delivery; 45.60% of the participants commended the internet speed at their institutions to be average, 34.43% speed below average, 14.71% speed above average, and 5.26% no internet access (Fig. 6). 56.76% were ready and have strategies for the incorporation of digital technology in the delivery of the courses, 30.79% on special occasions, 11.22% don’t think of any strategies, whereas 1.23% not applicable, perhaps in a traditional teaching and learning settings (Table 3).

Cost wise, the participants considered that the cost of licensing of the digital technologies or software is a factor that makes it difficult to use it in delivery of the courses, with 42.40% agreeing that it is total barrier, 30.81% sufficient barrier, while on the other hand, 22.28% thinks is a little factor, and 4.51% not a barrier (Table 4).

Practically, from the responses we received from the survey (*n* = 1576), we filtered out those participants who have completed the survey, by leaving out those that started but did not finish answering the survey. This resulted to a total number of *n* = 1071 responses. Also, considering the research representative target countries which we selected by taking into account the main regions in LATAM and primary focus of the educational, commercial, and financial investors (IDB, 2019; LASPAU, 2020; Microsoft, 2020; TEC, 2020a), which covers the large margin of the region’s population with major economies and top HEIs that are part of the Ibero-American association for distance higher education (AIESAD, 2020) in the emerging fields of TEL (Brunner & Ferrada, 2011; OECD, 2015); we further filtered the data sample to constitute only the nine main countries of our focus as follows: Argentina (*n* = 138), Brasil (*n* = 14), Chile (*n* = 37), Colombia (*n* = 130), Costa Rica (*n* = 26), Ecuador (*n* = 46), México (*n* = 372), Perú (*n* = 107), and Uruguay (*n* = 4) leaving out other countries that have responded to the distributed survey. Consequently, this resulted in a total number of *n* = 874 sample utilized throughout the research investigation and descriptive analysis of this study. To validate the adequate sample size needed to represent the LATAM population, we assume that half (50%) of the respondents should have either used technology for teaching, or have a technology training program in place in their different settings to give us maximum variability. To establish this, we assumed a 95% confidence level with a margin of error of ± 5%. Meaning that there could only be a 5% chance of our sample results differing from the target population. Thus, the estimate of the margin of error and confidence interval is given by 1/√*N* (Adam, 2020; Martínez-Mesa et al., 2014; Niles, 2006), where *N* is the number of participants in our sample (*n* = 874), which equaled to 0.03. This implies that if we eventually find that 50% of our participants have used digital technology for teaching or have a technology training program in place in their different HEI settings, then the actual proportion of the LATAM population we targeted could only vary by ± 3% (0.03). Interestingly, 52% of the participants claim to have used digital technologies for teaching/learning purposes, whereas ~ 79% indicated that their educational institutions’ have an Information and Communication Technology strategies or technology training program in place in their different HEI settings (see: Fig. 5 and Section 4). Moreover, going by the Cochran’s formula (Cochran, 1977) for calculating an ideal sample size, given the desired level of precision (margin of error of ± 5%) and 95% confidence level (where Z value is 1.96), and the estimated proportion of the attribute represented in our target population (50%), where *p* = 0.5. We note that statistically, the accepted adequate sample size for our study is approximately 385 in comparison to a total of *n* = 874 sample we have used/analyzed. Therefore, we strongly believe that there was enough data sample and variables/constructs to investigate or analyze in order to answer the research questions.

### Data analysis and results

The data analysis of this study was performed by considering the following set of constructs and hypotheses:

- For the qualitative approach, we performed a Text mining analysis (sentiment/emotional valence) to determine the top terms that the participants used to describe the use/challenges of digital technologies for teaching and learning in LATAM, and how the top most frequent terms are correlated or differ by the countries. This includes determining the implication or levels of impact of the emotional valence (terms quantification) in respect to the use of digital technology for teaching and learning in the region.
- In the quantitative approach, we applied a Kruskal–Wallis H-statistic to determine influential factors that the participants deem pertinent towards the reach, barriers, and bottlenecks in use of digital technologies in LATAM, and whether there may exist significant differences among the countries by considering the different items/constructs. Our hypothesis for testing whether there may exist a statistical significance/differences amongst the selected countries considering the items we have grouped based on the constructs (reach, barrier and bottleneck) was; *if* the variation, determined through the p-value or significance levels, is less than 0.05 (*p* < 0.05), *then* we assume there might be differences between the selected countries, and thus, we then subsequently analyze the individual countries using a Post-Hoc test to determine where the significant differences may lie (H_1_), *else if* the significant levels are greater than 0.05 (*p* > 0.05) *then* we can potentially reject the alternative hypothesis (H_1_), and statistically assume that there may not be differences in the reach, barriers and bottlenecks to use of digital technology for teaching and learning in the LATAM countries or region (H_0_).
- Finally, we evaluated the implications of both the statistical significances/differences in the findings by countries, evaluated the impact and implications of the top most used terms in the data by the participants and intensities of the different terms, and then provide an empirical discussion of the results.

#### Qualitative analysis

The study applied the Text mining technique (qualitative approach) to analyze opinions (viewpoints) given by the participants with regards to the current state-of-the-art, challenges, and implications of the use of digital technology for teaching and learning in HEIs in LATAM. Text mining is one of the techniques that is now being applied within the educational domain to analyze different textual (qualitative) datasets such as digital notes, formal documents, e-mails, chat messages, online discussion forums, comments or feedbacks received from the teachers and students, as utilized in this study (Hernández-Lara et al., 2021; Litman & Forbes-Riley, 2004; Mohammed et al., 2021; Okoye et al., 2020; Ortigosa et al., 2014; Tseng et al., 2018).

In this study, we applied the method (text mining) to determine the main patterns and/or frequency of terms perceived or used by the faculties to describe the way digital technologies has been used to facilitate the teaching and learning processes in HEIs in LATAM. Practically, we analyzed the comments (*n* = 874) provided by the participants in response to the question “In your opinion, what are the obstacles or challenges to using digital technologies for teaching–learning in higher education in LATAM?” (see Appendix 1) given to them in the survey in the form of an open-ended question.

**Fig. 7:**
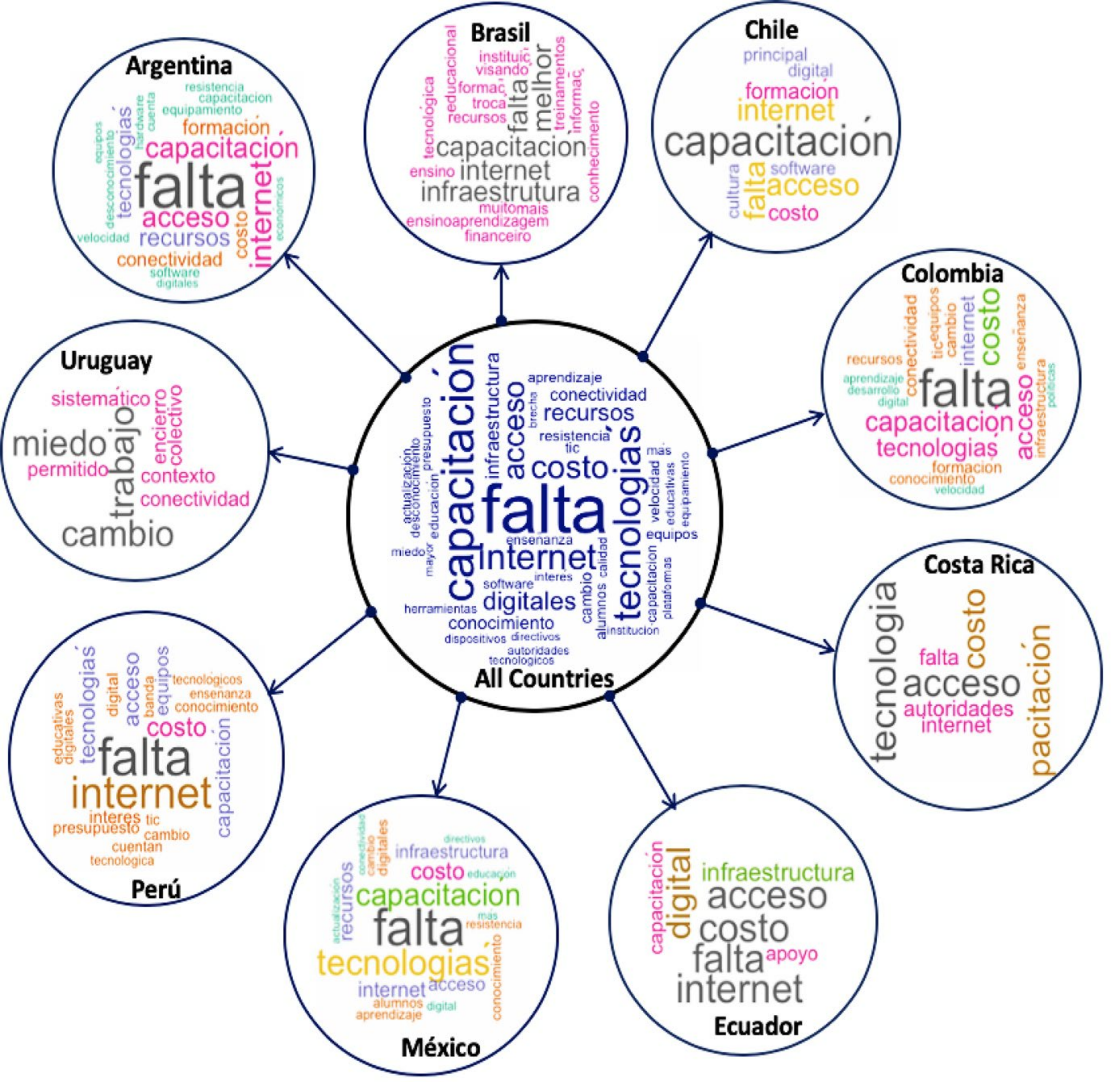
WordCloud of the top most frequent terms used by the participants to describe the use of digital technologies for teaching and learning in LATAM broken down by Country (the most common terms are listed in Fig. 8)

**Fig. 8:**
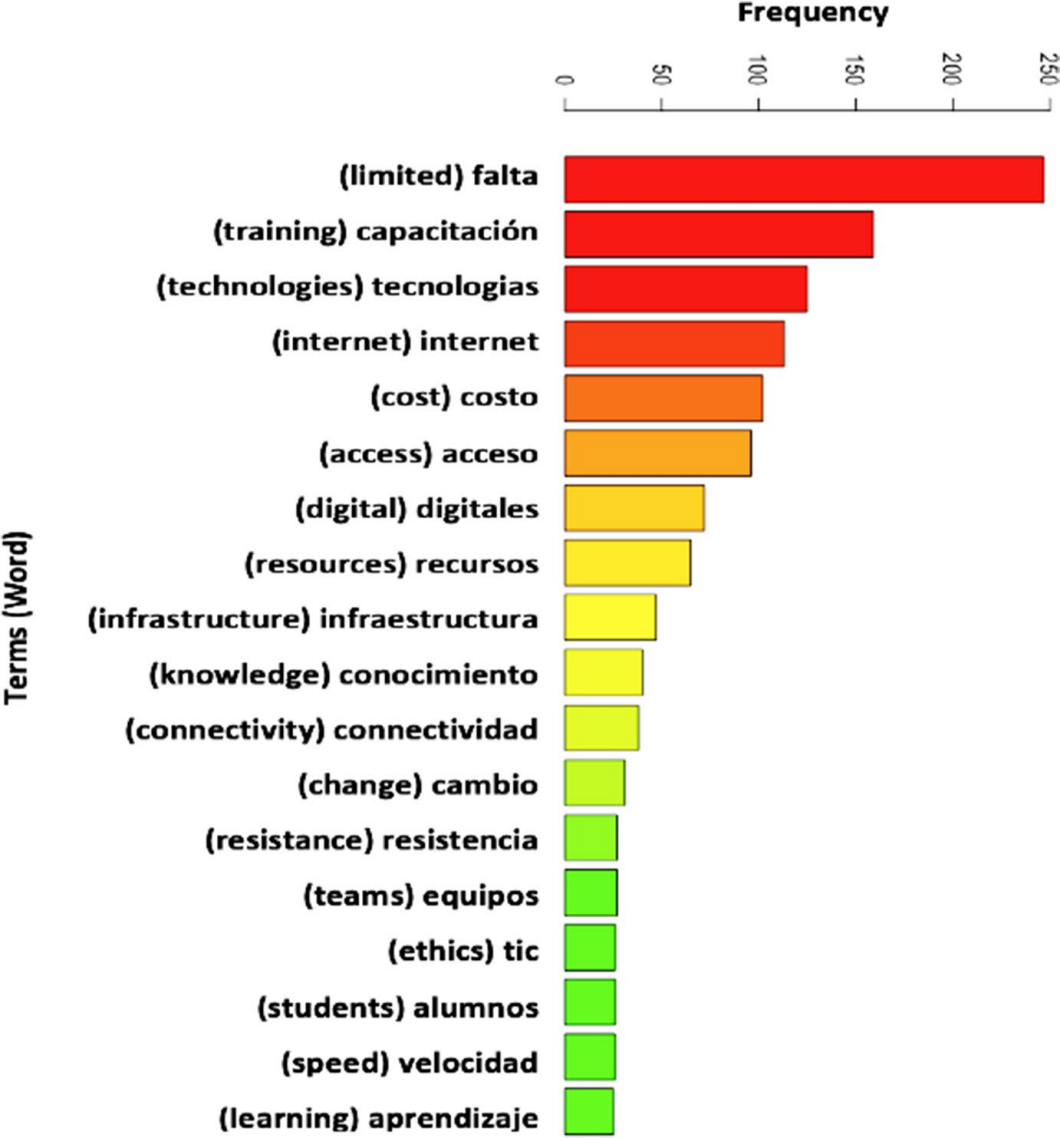
Chart representing the top most frequent used terms for the collective countries based on Fig. 7

Technically, we applied the Text mining method in R statistics (RStudio, 2018) to determine, first, the topmost frequent words (terms) used by the participants to describe the use of digital technologies in the various HEIs and country context. To do this, we created a corpus (library of words) of the different comments provided by the participants in the dataset that enabled us to identify the most frequent terms (see: Figs. 7, 8, and Appendix 2). Also, we splitted the data according to the nine participating countries, as shown in Figs. 7 and 8 (with full details about the individual countries most frequent terms provided in Appendix 2) to select the top five most frequent used terms for each individual country, and to further check how the terms were correlated/associated and/or differ as reported in Table 5.

**Table 5:** Correlation of terms or association analysis for the top five most frequently used words broken down by Country

Correlation of Terms broken down by Country									
Freq. Term	Argentina (*n* = 138)	Brazil (*n* = 14)	Chile (*n* = 37)	Colombia (*n* = 130)	Costa Rica (*n* = 26)	Ecuador (*n* = 46)	Mexico (*n* = 372)	Peru (*n* = 107)	Uruguay(*n* = 4)
Limited (Falta)	Recursos (0.35) Economicos (0.33) Política (0.23) Insumos (0.23)	Preparo (0.68) Aprendizagem (0.68) Tecnológica (0.68) Infraestrutura (0.42)	Digital (0.41) Estímulo (0.38) familiarizarse (0.38) conocimiento (0.38)	Educación (0.36) Claridad (0.33) Pedagogicos (0.33) Formación (0.24)	Conocimiento (0.35) Conectividad (0.35) Capacitación (0.13) Preparer (0.55)	Tecnológica (0.50) Politicas (0.35) Competencias (0.21) Capacitacion (0.21) Internet (0.28)	Tecnológicos (0.19) Capacitación (0.19) Recursos (0.19) Innovación (0.17)	Conocimiento (0.36) Capacidad (0.16) Vision (0.16) Tics (0.16)	No Correlated Term
Training (Capacitación)	Falta (0.26) Infraestructura (0.21) Accesos (0.20) Cuesta (0.20)	Política (0.68) Acesso (0.68) experiências (0.68) qualidade (0.68)	Calidad (0.29) Costumbre (0.29) Licenciamiento (0.2) Resistencia (0.14)	Reconocimiento (0.24 resistencia (0.10) Faltas (0.24) Institucional (0.24)	Conocimientos (0.2) Recursos (0.41) Utilizada (0.41) Falta (0.13)	Académicas (0.56) Efectiva (0.56) Investigación (0.56) Obligacione s(0.56) Costo (0.13)	Falta (0.19) Motivación (0.16) Infraestructura (0.12) Tradicionalista (0.11)	Resistentes (0.32) Acceso (0.13) Costo (0.21) Falta (0.19)	No Correlated Term
Access (Acceso)	Softwares (0.33) Tecnológicos (0.27) Internet (0.35) Computadoras (0.19)	No Correlated Term	Tecnologiaśas (0.47) Internet (0.38) Recursos (0.38) Calidad (0.38)	Internet (0.27) Tics (0.28) Aprendizaje (0.22) Económicos (0.16)	Internet (0.66) Servicios (0.37) Aprendizaje (0.37) Plataformas (0.37)	Internet (0.57) Politicas (0.35) Formación (0.21) Tecnológicos (0.35)	Internet (0.36) Tecnológicas (0.36) Herramientas (0.30) Ayudan (0.16)	Equipos (0.39) Capacitacion (0.33) Asesoria (0.30) Economicas (0.30)	No Correlated Term
Resources (Recursos)	Falta (0.35) Dificultades (0.32) Accesos (0.23) Económicos (0.33)	Financeiros (1)	Internet (0.38) Acceso (0.38) Estudiantes (1)	Suficiente (0.57) Institucional (0.40) Limitacion (0.40) Adquisición (0.40)	Financieros (0.69) Infraestructura (0.69) Costos (0.23) Capacitación (0.23)	Interner (0.57) Plataformas (0.35) Tecnologías (0.21)	Económicos (0.32) Falta (0.19) Infraestructuras (0.1) Limitación (0.16)	Suficientes (0.70) Educativos (0.70) Tecnológicos (0.34) Contenidos (0.49)	No Correlated Term
Internet (Internet)	Velocidad (0.40) Servicio (0.40) Obstaculo (0.25) Conexión (0.18)	Melhor (1) Acesso (0.68) Qualidade (0.68) capacitaç̃ (0.68)	Acceso (0.40) Computadores (0.38) Calidad (0.38) Velocidad (0.38) Recursos (0.38)	Aprendizajes (0.30) Servicios (0.20) Velocidad (0.28) Acceso (0.27)	Acceso (0.66) Costosas (0.55) Plataformas (0.55) Paradigmas (0.55) Antiguos (0.55)	Acceso (0.57) Plataformas (0.30) Falta (0.28) Permitan (0.35) Políticas (0.39)	Acceso (0.36) Velocidad (0.33) Calidad (0.24) Disponible (0.22)	Servicio (0.42) Accesiblidad (0.38) Lento (0.24) Acceso (0.20)	No Correlated Term

Cor limit = between 0 to 1 where 0 represents 0% and 1 represents 100% likelihood of the individual terms associated with the corresponding freq. term by country

Figure 8 is an overall chart representing the top most frequent terms used by the participants to describe the challenges to use of digital technologies for teaching and learning in HEIs across LATAM (i.e., for all countries). Further details (chart) about the most frequent terms for each of the individual countries are provided in Appendix 2. It is noteworthy to mention that we have implemented in the data cleaning process and normalization of the data; the three languages (English, Latin American Spanish, and Portuguese) in which the data sample have been collected before creating the TermDocumentMatrix table for the text analysis process. For example, removing of the punctuation marks and stopwords for 'english', 'spanish', and 'portuguese' in the R program.

Furthermore, the correlation of terms analysis that we performed to determine the association of the different terms in the TermDocumentMatrix (dataframe), or how the factors are related (correlated) is reported in Table 5.

As gathered in Table 5, the most frequently used term to describe challenges in use of digital technologies for education by the selected LATAM countries includes “limited”, “training”, “access”, “resources”, and “internet”, otherwise attributed to the reach, barriers, and bottlenecks to the use of digital technologies in education in the context of this study, respectively. The results purportedly suggests that the participants considered the aforenoted factors as the main challenges or critical for effective teaching and learning processes in LATAM. In other words, if HEIs across LATAM would focus on addressing the identified issues of lack of training, lack of infrastructures and resources, and ensure access to the internet. Not only will the so-called stakeholders (teachers, students, staffs) benefit in terms of the use of digital technologies to foster education, but also, this would play a vital role in support of the educational/learning goals of the educators, commercial, and financial investors in LATAM, at a wider scale (IDB, 2019, 2020; LALA, 2020; LASPAU, 2020; Microsoft, 2020; TEC, 2020a). Moreover, this result (correlation of terms—Table 5) also aligns with the set of 33 barriers found by Mercader and Gairín (2020), and the several socio-technical factors that have been identified to potentially slow down or affect the concrete application/performance (bottlenecks) of the use of digital technologies for teaching and learning in LATAM (Bezanilla et al., 2019; LALA, 2020).

Furthermore, this study also deemed it important to determine the impact (intensity levels) of the comments provided by the participant in respect to the use of digital technology for teaching and learning in LATAM. We applied the *emotional valence* (sentiment analysis) method in R which focuses on measuring (through polarization or term quantification) the intensities of the individual comments provided by the participants, by extracting and assigning a score to each word or term found in the comments that can be used to express an emotion, and then quantifying the comments according to the number of scores (emotional terms) found in each case or comment.

To do this, we applied the *get_nrc_sentiment* function in R to extract the different (emotional valence) scores for each of the comments broken down by country. Typically, the get_nrc_sentiment functions by obtaining and quantifying (polarization) the intensities of the different words/terms that can be used to express emotion in the texts using the positive ( +), neutral (0), and negative (-) values (Litman & Forbes-Riley, 2004) to represent each relevant word it finds in each case. In Table 6, we present results of the method and scores for the comments (*n* = 874) across the dataset, considering all the selected LATAM countries. As gathered in Table 6, we showed some examples of the (emotional valence) scores for the first 228 comments in the dataset (*n* = 874), whereby; The Comments column, [1] to [210], represents the id of the individual comments in each case within the corresponding row (Table 6).

**Table 6:** Fragment of the Emotional Valence scores for the different comments provided by the participants towards the use of digital technologies for teaching and learning in LATAM

Emotional Valence scores for All the LATAM countries	
Comments	Valence Score
[1]	0 0 1 0 1 0 0 0 0 0 0 0 1 0 0 0 0 0 0
[20]	-1 0 0 0 0 0 0 0 0 0 0 0 0 3 0 0 1 0 0
[39]	0 -1 1 0 1 0 0 0 0 0 0 0 0 0 2 -1 0 0 0
[58]	-1 0 0 0 -1 0 0 1 0 0 0 0 0 0 0 0 -1 0 1
[77]	0 0 0 0 -1 0 0 0 0 0 -1 0 0 0 0 2 0 1 0
[96]	0 0 0 0 0 -1 0 0 1 0 0 0 -1 0 0 0 0 0 0
[115]	1 -1 0 0 0 0 0 0 0 0 0 0 0 1 0 0 0 0 0
[134]	1 0 0 0 0 0 0 0 0 0 0 0 0 0 0 0 0 0 0
[153]	0 0 0 0 0 0 0 0 0 0 0 0 0 0 0 0 0 1 0
[172]	0 0 0 0 0 0 0 -1 0 0 0 0 0 0 0 0 0 0 0
[191]	0 0 0 1 -1 0 0 0 0 0 0 0 0 0 0 0 0 0 0
[210]	0 0 0 0 0 0 0 0 0 0 0 0 0 0 0 -1 0 0 0

Min = -2, Median = 0.00, Mean = 0.04, Max = 3

values = positive ( +), neutral (0), negative (-)

**Fig. 9:**
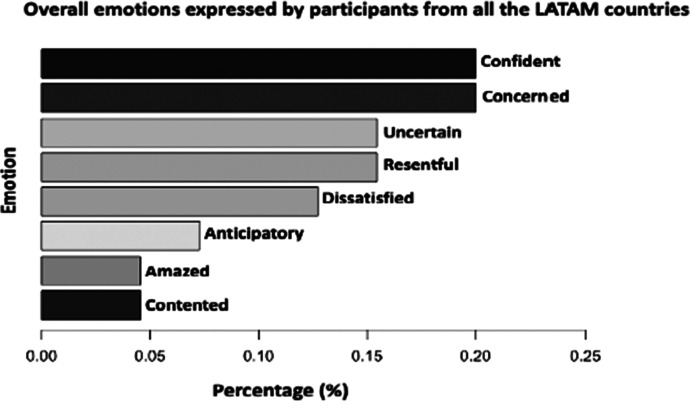
Chart representing the overall emotions (classification) expressed by the participants across the data (*n* = 874)

Table 7 is a summary of the Min, Median, Mean, and Max scores we have found for each of the representative countries, whilst Fig. 9 is an overall chart representing the summary of the different emotions (classifications) expressed by the participants across the data based on the educational research domain (Kort et al., 2001; Litman & Forbes-Riley, 2004; Okoye et al., 2020; Shen et al., 2009; Tian et al., 2010). Full definition or details about the different emotions (classifications) expressed by the participants broken down by country is provided in Appendix 3.

**Table 7:** Summary of Emotional Valence scores expressed by the participants broken down by Country

Emotional Valence scores				
Country	Min	Median	Mean	Max
Argentina	-1.00	0.00	0.06	1.00
Brasil	0.00	0.00	0.00	0.00
Chile	0.00	0.00	0.13	2.00
Colombia	-1.00	0.00	0.07	2.00
Costa Rica	-1.00	0.00	-0.09	0.00
Ecuador	0.00	0.00	0.13	1.00
México	-1.00	0.00	0.07	3.00
Perú	-2.00	0.00	-0.05	1.00
Uruguay	0.00	0.00	0.00	0.00
All Countries	-2.00	0.00	0.04	3.00

Max-positive ( +), Min/Max-neutral (0), Min-negative (-) values

The comments with positive valence ( +) scores represent an attractive emotion, whilst the negative (-) scores signify an aversive emotion. The zeros represents comments that was classified as neutral (0) with no emotional terms found, thus, no word(s) which can be used to express emotions were found in those cases.

The results, reported in Tables 6 and 7, shows that the valence scores (i.e., min and max) for the analyzed countries ranged between -2 to 3, respectively. The most positive emotions (Max) was expressed by countries such as México, Chile, and Colombia (see: Table 7), while Perú have shown the least emotion (Min). Interestingly, the aforenoted observations, when triangulating the results of this study, occurred to align with the results of the quantitative analysis (see Sections 3.3.2 and 4); therein we also found under the analyzed constructs (see: Table 8) that Mexico and Peru appeared to share a substantial difference, including Chile and Colombia. Specifically, as gathered in Table 8, those differences were observed for how prepared the participants felt to incorporate digital technology for teaching and learning purposes, how well the users know about digital technology and are competent in applying the digital technologies to the courses or disciplines they teach, and whether there are provisions or any technological platform used to manage the students’ learning in their different HEI settings (see: Fig. 5 and Table 3). The implications of these observations could be that in LATAM, HEIs in such regions such as Perú are yet to fully profit from the vast benefits of using the digital technologies to support the teaching–learning processes which we have discussed in detail in the Implication of this Study section (Section 4.1).

**Table 8:** Result of the Kruskal–Wallis H-test considering the research constructs: reach, barrier, and bottleneck

Kruskal–Wallis test statistics based on the constructs: Reach, Barrier, Bottleneck				
Construct	Question(Q)	Description	r (X^2^)	p-value
Reach (alcance)	18	Which of the following technologies do you know that your educational organization explores, is developing or has implemented today? *(¿Cuál de las siguientes tecnologías conoce que su organización educativa explora, esta desarrollando o ha implementado al día de hoy?)*	19.453	0.012*
	19	For what purpose (s) do you use digital technology in your courses? *(¿Con qué propósito(s) emplea tecnología digital en sus cursos?)*	9.4526	0.305
	24	From the following criteria choose those that you take into account to incorporate a digital tool in your courses *(De los siguientes criterios elija aquellos que toma en cuenta para incorporar una herramienta digital en sus cursos)*	5.9756	0.650
	17	What are the digital technologies that students require to use for their courses? *(¿Cuáles son las tecnologías digitales que requiere que los estudiantes usen para sus cursos?)*	13.381	0.099
	25	Do you have strategies for incorporating digital technology in the delivery of your courses? *(¿Tiene estrategias para la incorporación de tecnología digital en la impartición de sus cursos?)*	10.135	0.2557
	26	Do you use digital technology to collect, analyze, and interpret data on student progress? *(¿Utiliza la tecnología digital para recopilar, analizar e interpretar datos sobre el progreso de los estudiantes?)*	3.0541	0.930
Barrier (barrera)	16	Do you think that the digital tools available in your educational organization are useful for teaching in the courses you teach? *(¿Considera que las herramientas digitales disponibles en su organización educativa resultan útiles para la enseñanza en los cursos que imparto?)*	14.083	0.079
	20	How prepared do you feel to incorporate digital technology for teaching–learning purposes? *(¿Qué tan preparado se siente para incorporar tecnología digital con fines de enseñanza-aprendizaje?)*	16.882	0.031*
	21	How well do you know about digital technology applicable to the courses or discipline you teach? *(¿Qué tanto conoce de tecnología digital aplicable a los cursos o disciplina que imparte?)*	16.672	0.033*
	22	Do you consider that the cost of licensing digital technology is a factor that makes it difficult to use it in your courses? *(¿Considera que el costo de licenciamiento de tecnología digital es un factor que dificulta utilizarla en sus cursos?)*	10.535	0.229
	23	To what extent is the possibility of error or failure of digital technology a factor that you consider when deciding whether or not to use it in your courses? *(¿En qué medida la posibilidad de error o fallo de la tecnología digital es un factor que considera al momento de decidir si la utiliza o no en sus cursos?)*	14.422	0.071
	37	In your opinion, what are the obstacles or challenges to using digital technologies for teaching–learning in higher education in LATAM? *(En su opinión ¿Cuáles son los obstáculos o retos para usar tecnologías digitales para la enseñanza-aprendizaje en la educación superior en América Latina?)*	-	-
Bottleneck (cuello)	27	How would you catalog access to the Internet in your educational organization? *(¿Cómo catalogaría el acceso al Internet en su organización educativa?)*	18.58	0.017*
	28	Does your educational organization have an Information and Communication Technologies services and support area? *(¿Su organización educativa cuenta con un área de servicios y soporte de Tecnologías de Información y Comunicación?)*	22.421	0.004*
	29	Does the Department of Information and Communication Technologies report to the highest authority of your educational organization? *(¿El departamento de Tecnologías de Información y Comunicación reporta a la máxima autoridad de su organización educativa?)*	9.0295	0.339
	30	Is any technological platform used to manage student learning in your organization? Example: LMS—Learning Management System such as Canvas, Blackboard, Google classroom, etc.) *(¿Se utiliza alguna plataforma tecnológica para la gestión del aprendizaje del alumno en su organización? Ejemplo: LMS—Learning Management System como Canvas, Blackboard, Google classroom, *etc*.)*	37.208	0.000*
	31	What is the degree of use of the LMS platform for the management and teaching of your courses? *(¿Cuál es el grado de uso de la plataforma LMS para la gestión y enseñanza de sus cursos?)*	8.9616	0.345
	32	Do you think that the educational organization in which you work has a vision of how students and teachers should use digital technology to improve teaching and learning? *(¿Considera que la organización educativa en la que labora tiene una visión de cómo los estudiantes y los profesores deberían utilizar la tecnología digital para mejorar la enseñanza y el aprendizaje?)*	26.45	0.001*
	33	Is there a training plan on the use of educational technology in your educational organization? *(¿Existe un plan de capacitación sobre el uso de tecnología educativa en su organización educativa?)*	20.336	0.009*
	34	How effective is the training plan on the use of educational technology? *(¿Qué tan efectivo es el plan de capacitación sobre el uso de tecnología educativa?)*	8.9848	0.343
	35	Does the educational organization where I work promote spaces to discuss and plan in a collegial way about the use of digital technology in teaching–learning? *(¿La organización educativa en donde laboro promueve espacios para discutir y planificar de manera colegiada sobre el uso de tecnología digital en la enseñanza-aprendizaje?)*	13.792	0.087
	36	Does the educational organization where you work give you any incentive or recognition for developing educational innovation projects using digital technology? *(¿La organización educativa en donde labora le otorga algún incentivo o reconocimiento por desarrollar proyectos de innovación educativa usando tecnología digital?)*	8.7916	0.360

Significant level = (*), *p*-value ≤ 0.05, Confidence Interval (CI) = 95%, *df* = 8 for nine groups of countries

It is also noteworthy to mention, as shown in Fig. 9, that while the participants trusts or are confident (approximately 20%) that the digital technologies and literacy skills are important, and integral towards achieving an effective teaching and learning processes across the several HEIs in LATAM. At the same time, they are equally susceptible or concerned (~ 20%) about its effectual use and ample implementation for such (educational) purposes considering the different challenges or influential factors we have reported and discussed earlier (see: Figs. 7, 8, Table 5, and Background Information). Moreover, from the results in Fig. 9, it can be said that the low percentages (~ 5%) of participants that indicated surprise (amazed or contented) in their response, could mean that the use of digital technologies for teaching and learning (digitized-education) have shifted from being just an effective form of delivery of education, perhaps thanks to the nudge or lessons from the recent global pandemic, but also, on the other hand, is revolutionizing or transforming the way education and its outcomes are being indulged or perceived. Thus, this can explain why the participants, or yet educators, are still to overcome the many challenges (reach, barriers, bottlenecks) with TEL-based education or fully gain from its vast benefits and application in the region, as found in both the results of this study (Figs. 7, 8, Table 5), and review of the current state-of-the-art/literatures (see: Background Information). In practice, those findings and observations means introduction of suitable practices by the concerned stakeholders (educational policymakers, curriculum designers, teachers training and skills, investors, and partnerships) that purportedly includes educational components or frameworks that cater for thoughtful and introspective transformation of the current (education) ecosystem and routines across the region (LALA, 2020; Selwyn, 2021; UNESCO, 2021a, c).

#### Quantitative analysis

For the quantitative analysis, we conducted a Kruskal–Wallis H non-parametric statistical test (Frey, 2018) in R statistics (RStudio, 2018), to determine the significance (impact) of the different group of variables/items we classified according to the construct (Reach, Barrier, and Bottleneck) by taking into account the different countries, as stated in the research question and hypothesis. It is important to mention that the study have used the Kruskal–Wallis test to analyze the data due to its powerful, distribution-free, and ability to produce statistically significant results without being affected by outliers (Derrick et al., 2020). Also, we have applied the method (Kruskal–Wallis) because the variables we analyzed were in ordinal data format derived from the Ranked Likert-scale and multiple-choice questions (see: Table 1 and Appendix 1). The result of applying the method and statistics based on the constructs (Reach, Barrier, and Bottleneck) is reported in Table 8.

As gathered in Table 8, we found that there was a significant difference (*p* ≤ 0.05) between some of the different items and classifications (constructs), i.e., Reach (alcance), Barrier (barrela), and Bottlenecks (cuello), which we have analyzed by considering the countries. Therefore, we can statistically accept the alternative hypothesis (H_1_) as stated in the research question (see: Rationale of this study), and assume that there exist differences between the countries. Statistically, the differences were observed for the following items (Question): 18, 20, 21, 27, 28, 30, 32, 33 (see: Table 8 for individual Items description). Whereby:

- Item 18 was significant for the Reach (alcance) construct
- Items 20 and 21 for Barrier (barrera), and
- Items 27, 28, 30, 32, and 33 for Bottleneck (cuello), respectively

Furthermore, given that significant differences (see above Items of which *p* ≤ 0.05) were observed for the countries, we performed a post-hoc test in order to determine where the differences may lie according to the individual countries, taking into account the significant results in Table 8.

To do this, we conducted a Kruskal–Wallis multiple comparison dunn.test (Elliott & Hynan, 2011) adjusted using the "bonferroni" method in R, to determine the differences by country. The result of the post-hoc test is presented in Table 9. It is important to mention that we have reported only the significant values for the post-hoc test in Table 9. Full detail and statistics about the individual countries and the post-hoc comparison are provided in the Appendix section (Appendix 4).

**Table 9:** Post-hoc test considering the variables (constructs) that were pertinent to the use of digital technologies for teaching–learning in HEIs in LATAM by country

Post-hoc = Dunn (1964) Kruskal Wallis multiple comparison test, p-values adjusted with Bonferroni method					
Construct	Question (Q)	Comparison	Z	Unadjusted p-value	Adjusted p-value
Reach (alcance)	Q18	Colombia – Perú**	3.47414	0.00051	0.01844
		México – Perú**	3.66743	0.00024	0.00881
Barrier (barrera)	Q20	Argentina – Perú*	3.09325	0.00197	0.07127
		México – Perú**	3.67504	0.00023	0.00856
	Q21	Argentina—Perú*	2.85531	0.00429	0.15477
		México—Perú**	3.61557	0.00029	0.01078
Bottleneck (cuello)	Q27	Chile—México*	2.77124	0.00558	0.20103
		Colombia—México*	2.34516	0.01901	0.68467
		México—Perú*	-2.66068	0.00779	0.28073
	Q28	Argentina—Chile**	-2.44819	0.01435	0.51686
		Argentina—Colombia**	-3.73620	0.00018	0.00672
		Argentina—Costa Rica*	-2.90913	0.00362	0.13047
		Argentina—México**	-3.56961	0.00035	0.01287
		Argentina—Perú*	-2.24856	0.02454	0.88344
	Q30	Argentina—Chile**	-3.11681	0.00000	0.06581
		Argentina—Colombia**	-4.00836	0.00000	0.00220
		Argentina—México**	-4.02506	0.00000	0.00205
		Chile—Perú**	3.25203	0.00000	0.04125
		Colombia—Perú**	4.08424	0.00000	0.00159
		México—Perú**	4.05093	0.00000	0.00183
	Q32	Brasil—Chile*	2.63978	0.00829	0.29864
		Brasil—Ecuador*	2.64211	0.00823	0.29660
		Chile—México*	-3.02394	0.00249	0.08982
		Colombia—México*	-2.50936	0.01209	0.43541
		Ecuador—México**	-3.19547	0.00139	0.05025
		México—Perú*	2.70152	0.00690	0.24847
	Q33	Argentina—Colombia**	-3.27486	0.00105	0.03805
		Brasil—Colombia*	-2.32250	0.02020	0.72740
		Colombia—Ecuador*	2.67400	0.00749	0.26982
		Argentina—México*	-2.33231	0.01968	0.70863
		Brasil—Uruguay*	-2.36463	0.01804	0.64972

*p* ≤ 0.05 Significant Levels = highly sig. (**), slightly sig. (*), see: Table 8 for description of the questions/items (Q)

## Discussion

The analysis and results of this study was based on the use and impact of digital technologies upon the teaching and learning processes in HEIs in LATAM. In the empirical study, which we performed with emphasis on Colombia, Brazil, Mexico, Argentina, Chile, Peru, Costa Rica, Ecuador, and Uruguay, based on the main focus of the educators, commercial, and financial investors in LATAM (IDB, 2019, 2020; LASPAU, 2020; Microsoft, 2020; TEC, 2020a). We note that some upholding factors based on the studied variables (see: Tables 1, 8, 9) appeared to be significantly relevant when considering the reach (alcance), potential barriers (barrera), and bottlenecks (cuello) in use of digital technologies for teaching and learning across the nine selected countries.

When considering the *reach* (alcance) construct, both the qualitative and quantitative results shows that the use and implementation of digital technologies for teaching and learning purposes in the HEIs is important, as well as, challenging for the users (see: Figs. 7, 8, 9 and Tables 5, 7, 9). Although, it can also be said that the challenges and differences were mainly observed for countries such as Peru, who have significantly shown to express limited or lack of digital resources as one of the main challenge as opposed to countries like Colombia and Mexico (*p* < 0.05) (see: Table 9).

Under the *barrier* (barrera) construct; the authors note that when asked how prepared the stakeholders feel to incorporate digital technology for teaching and learning purposes (Item 20; Table 9), Mexico and Peru appeared to have a higher significant difference (Unadj. *p* = 0.00023, Adj. *p* = 0.00856). Whereas, Argentina and Peru shared a slight difference (Unadj. *p* = 0.00197, Adj. *p* = 0.07127). By slight difference we refer to the results (see: Table 9) in which the Unadjusted p-values were less than 0.05 (*p* < 0.05), while the Adjusted p-values are greater than or equals to 0.05 (*p* ≥ 0.05). The aforenoted results suggests that countries who are classified as high-tech or have greater access to digital technologies, such as Mexico, tends to share distinctive opinion or challenges in comparison to the low access regions such as Peru. Although, this speculation has only been studied within the context of Mexico (Sánchez-Cruz et al., 2021), and from a global perspective (UNESCO, 2021a). Again, this may also explain the reason why Argentina shared a slight difference with Peru (Table 9). Interestingly, when asked how well the users know about digital technology and are competent in applying the digital technologies to the courses or disciplines they teach (Item 21; Table 9), Mexico and Peru again shared a significant difference (Unadj. *p* = 0.00029, Adj. *p* = 0.01078), whilst Argentina and Peru had a slight difference (Unadj. *p* = 0.00429, Adj. *p* = 0.15477). Interestingly, the pieces of evidence we found for the qualitative analysis (see: Table 7) also bring into line the same aforementioned affirmations.

Likewise, when considering the *bottleneck* (cuello); we found that there were slight differences (Unadj. *p* < 0.05, Adj. *p* ≥ 0.05) for Chile-Mexico, Colombia-Mexico, and Mexico-Peru, when asked how the users would rate the access to internet in their various establishments (Item 27; Table 9). When we analyzed the level of ICT support that is being provided for the stakeholders by the different HEIs (Item 28; Table 9), Argentina-Chile, Argentina-Colombia, Argentina-Mexico, shared a significant difference (Unadj. and Adj. *p* < 0.05), which suggests that HEIs or the faculties in countries such as Chile, Colombia, and Mexico, may tend to have more access to technological support than their counterparts in Argentina. Argentina-Costa Rica, and Argentina-Peru, came out with a slight difference (Unadj. *p* < 0.05, Adj. *p* ≥ 0.05) which suggests that challenges with the level of ICT support received by faculties in those countries are slightly different or yet close to being the same. Also, there were significant differences (Unadj. and Adj. *p* < 0.05) between Argentina-Colombia, Argentina-Mexico, Chile-Peru, Colombia-Peru, Mexico-Peru, and a slight difference between Argentina-Chile (Unadj. *p* = 0.00000, Adj. *p* = 0.06581), when asked if there are technological provisions or platforms used to manage the students’ learning process in the different HEIs’ settings (Item 30; Table 9). In the results, we also found that when asked about the infrastructures and use of digital technology to improve the teaching and learning process for the stakeholders (Item 32; Table 9), Ecuador-Mexico, Brasil-Ecuador, Chile-Mexico, Colombia-Mexico, and Mexico-Peru, all presented a slight difference (Unadj. *p* < 0.05, Adj. *p* ≥ 0.05) with the most noted significance being for Ecuador-Mexico (Unadj. *p* = 0.00139, Adj. *p* = 0.05025). Finally, we found a significant difference between Argentina-Colombia (Unadj. *p* = 0.00105, Adj. *p* = 0.03805), and a slight difference (Unadj. *p* < 0.05, Adj. *p* ≥ 0.05) between Brasil-Colombia, Colombia-Ecuador, Argentina-Mexico, Brasil-Uruguay, when asked if there is a training plan on the use of educational technology for the users across the different educational institution (Item 33; Table 9), and vice and versa.

To summarize the above points and observations, by considering the results of the three constructs we have analyzed (reach, barrier, bottleneck): it can be said that the main challenging factors and/or differences we have observed for the different countries has to do with the “bottleneck” construct (Table 9), which can be allied to issues in the use or adoption of the digital technologies for learning that causes the teaching–learning process to slow down or affects the practical application, performance, or adoption of the educational technologies for teaching and learning in the region (Raza, 2019). Furthermore, in the qualitative analysis (text mining) we found that majority of the positive scores (emotional valence) have been expressed by countries such as México, Chile, and Colombia (see: Table 7), while Perú showed the most minimum score. Indeed, such cross verifications, subsequent to data-triangulation of this study (qualitative vs quantitative), appears to support both the multiple causes we have found in our analysis that impacts the use of digital technologies for teaching and learning in LATAM (Fig. 7 and 9, Tables 7 and 9), and the pieces of evidence we have drawn from the literature (see: Background Information). The results reported in Tables 7 and 9 showed that Peru appeared to be the most affected country considering the three constructs: reach, barriers, and bottlenecks. Although, Argentina also significantly showed to be affected in terms of the bottleneck factors (Table 9).

Another interesting finding from the qualitative (text mining) analysis we conducted (Fig. 9), is the fact that while the participants were “confident” that the digital technology is relevant towards achieving an efficient and impactful teaching and learning process in HEIs in LATAM, they are equally “concerned” about the several challenges or effective use of the digital technologies for teaching and learning, especially considering the prominent factors we have highlighted in Figs. 7 and 8. For example, while the OECD (2015) notes that although a majority of the HEIs (65%) in LATAM believe that the teachers have the adequate training to develop e-learning contents, 26% believe they do not. With 41% of the HEIs endorsing the innate risk that such gap poses to the region (OECD, 2015). Interestingly, when looking into the main reasons behind the adoption and increase in demand for e-learning programs/technologies in the region, the OECD (2015) notes that 71% of the HEIs (both public and private) perceives the cumulative penetration of digital technologies in their respective settings or iterations as one of the main drivers. Whilst 50% highlights the need for incorporating the distance education or e-learning tools for fostering the modern-day education.

Overall, both results of our method (qualitative and quantitative analysis) indicates that the respondents put more emphasis on the lack or limitation of training, infrastructures and resources, and access to the internet or digital platforms (Figs. 7, 8; Tables 5, 8, 9, Appendix 2) as the main challenges to effective use of digital technologies for teaching and learning across the different HEIs in LATAM. Interestingly, the aforementioned challenges we have found also aligns with the barriers, and set of 33 obstacles found in the recent study of Laufer et al, 2021, and Mercader and Gairín (2020), respectively.

Theoretically, there has been several speculations in both the Educational and Scientific research, on whether institutional leaders, regulatory bodies, and faculties, would altogether embrace and benefit from the new educational innovations or EdTechs for teaching and learning. Practically, the answer is yes, as the many institutions have developed several innovative practices that will eventually advance the use of digital technologies in and for educational purposes (Boninger et al., 2019; Crawford et al., 2020; Garcez et al., 2021; Haruna et al., 2019; Hosseini et al., 2021; Martens et al., 2020; Méndez-Reguera & López, 2021; Mikheev et al., 2021; Muhaimin et al., 2020; Munro, 2018; Okoye, et al., 2021; Pettersson, 2020; Toit & Verhoef, 2018; UNESCO, 2021a, b). However, this is only sustainable if the different stakeholders (e.g., higher educational institutions, financial investors, government, and policymakers) will incorporate the crucial need for addressing the different challenges as identified in this study. For all intents and purposes, the pieces of evidence we drew from the literature and results of this study (see Sections 2 and 3.3), shows that TEL initiatives must be the strategic focus of the many institutions especially across the national margins/region of LATAM, to ensure that the use of digital technologies for teaching and learning are fast-tracked (LALA, 2020).

In that perspective, this study also, on the other hand, note that most HEIs are not readily prepared to face those challenges of adopting the new digital technologies and its underlying global reality and/or transformational benefits (Boninger et al., 2019, 2020; CONECTA, 2021a; Cuban, 2020, 2021; OECD, 2015, 2021). The lack of preparedness became patent when the universities and faculties were forced to shift to a completely-digital modality due to the recent Covid-19 pandemic (Aguilera-Hermida et al., 2021; Almaiah et al., 2020; Al-Maskari et al., 2021; Aristovnik et al., 2020; Armstrong-Mensah et al., 2020; Blackman et al., 2020; Burgess & Sievertsen, 2020; Crawford et al., 2020; Crick et al., 2020; Devkota, 2021; di Pietro et al., 2020; Ma et al., 2021; Mncube et al., 2021; OECD, 2020b, 2021; Oyedotun, 2020; Peres et al., 2020; Pokhrel & Chhetri, 2021; Reimers et al., 2020; Rogers & Shwetlena, 2020; Sánchez-Cruz et al., 2021; Seetal et al., 2021; UN, 2021; UNESCO, 2020; Viner et al., 2020). Besides, a larger part or reason for such lack of readiness may be attributed to the unavailability of infrastructures, digital literacy skills, or limited resources in the region (LALA, 2020; Sánchez-Cruz et al., 2021; UNESCO, 2021a, b).

As an example, from the demographic or descriptive statistics of the collected data which we have provided in the data sampling section (Section 3.2); the study found that approximately ~ 40% of the participants in the survey claimed that they don't have access to an internet connection or that it is very slow (34.43% speed below average, and 5.26% no internet access) (Fig. 6). This suggests that efforts regarding internet access in LATAM are still not sufficient. At the institutional level, an approximated ~ 21% of the professors commented that their institutions do not have a technology or IT support department, or do not know whether there is such support in place (17.83% do not have, 3.12% do not know) (Fig. 5). Although, 56.76% of the participants agreed to being ready and have strategies for incorporation of digital technology in the delivery of the courses. In addition, ~ 73% of the participants claimed that the cost of licensing and software is a barrier to the use of technology in their institutions, with 42.40% agreeing that it is total barrier, while 30.81% stated that it is a sufficient barrier (Table 4). Therefore, with those findings, this current study believes that entering a “digital transformation” will require specialized support from an institutional level or perspective that provides adequate equipment and training for the so-called stakeholders (teachers, students, staffs, etc.) (Boninger et al., 2019, 2020; CONECTA, 2021a; Cuban, 2020, 2021; Laufer et al, 2021; OECD, 2015, 2021; Okoye et al, 2021; Raza, 2019). Likewise, it is recommended to consider public policies that allow the HEIs to have access to funds specific to the acquisition of educational technologies and network infrastructures to meet those identified challenges or barriers (LALA, 2020; Selwyn, 2021; UNESCO, 2021a, c).

Furthermore, although majority of the faculties showed to have attained graduate studies, i.e., Doctorate and Masters (approximately ~ 70%) (Table 2), we further found that almost ~ 43% do not feel prepared to incorporate digital technology into their teaching practices and/or offered courses. Perhaps, this could largely depend on the socio-economic factors, for instance, as found by OECD (2015) where 51% of the quintile with the highest education shows to have access to a computer, and 29% to internet. With only 1% of those with the lowest education having access to computer, and/or literally no access to the internet (OECD, 2015).

We also observed in our study that 48% of the professors do not teach any online course, whilst 52% claim to have used digital technologies for teaching. Although, ~ 90% (58.97% totally agree, 31.38% agree) of the participants, on the other hand, claimed to have used digital technologies for teaching–learning purposes, or yet during their academic career (Table 3). Also, while a smaller proportion of the faculties surveyed (~ 21%) commented that their institutions do not have a training program to facilitate or support the use of technology for teaching or learning (17.83%), or do not know (3.12%) (Fig. 5). For the 52% that claimed to have used digital technologies for teaching, we found in a further follow-up study that a third-part (~ 32%) of them claimed that the programs are not effective. This suggests a significant gap in attaining digital transformation in the region, particularly in terms of socio-technical perspectives that could be addressed by promoting digital competences and knowledge acquisition through the culture of *educational innovation*. The current educational paradigm especially for teaching or learning relies on the balance of *soft-technological skills* and *discipline-specific expertise*. Therefore, the authors strongly believe that effective mechanisms or paradigms for developing new sets of skills is prerequisite in the training of new professors. Moreover, we greatly trust that *training effectiveness* and *continuous improvement* through some magnitudes of impact measurements and constructs should be included in the faculties’ development program.

The set of key conclusions, both from the evidences we drew from the literature review and results of this study, which can be applied by HEIs to advance the use of digital technologies in the different settings or contexts, particularly in alignment with the four goals for education (Goals 23, 24, 25, and 26) reinforced by the Lima Declaration (ECLAC, 2013), includes as follows:

- The perception of the surveyed faculties is that HEIs are challenged in their digital transformation at different dimensions. From resource allocation to effective faculty’ development and training programs, e.g., digital skills acquisition. There is a need to address those issues to avoid a broader educational gap in LATAM.
- It is recommended that HEIs take into consideration “techno-based” skills in the process of developing and attracting professors. For active faculties, it is essential to design training programs aligned with the digital world, and in collaboration with the government and the different companies’ educational initiatives.
- It is essential to create conditions that enable universities to have access to educational technologies and fund, in order help develop educational tools/models that are specially tailored to the region's realities and technological requirements. For instance, deployment of adequate infrastructures, networks and internet connection that enable those digital technologies to function (LALA, 2020; OECD, 2015, 2016, 2017; UNESCO, 2012). Besides, the digital infrastructures are essential for the stakeholders (e.g., teachers and students) to thrive in this new digital-savvy or unprecedented changes in the way learning takes place.
- There is an opportunity to reach a higher number of students, including quality of education and efficiency of learning, by developing both online and blended education across the region (Okoye et al, 2021; UNESCO, 2021b).

It is important to consider new and innovative ways of delivering education or knowledge acquisition/opportunities for the students. Moreover, when used effectively, digital technologies can provide better learning outcomes (Armstrong-Mensah et al., 2020; Pokhrel & Chhetri, 2021), and increase access to education or mobility, new opportunities for the students and faculties, and competitiveness within the global and labor market at large (UNESCO, 2020, 2021a).

### Implications of this study

This study can be allied to both (i) Research and pedagogical implications, (ii) Socio-cultural factors and impact, and (iii) Global or practical paradigms/practices, that may impact the use of digital technologies for teaching and learning, particularly in LATAM.

#### Research and pedagogical implications

Today, educational technologies are used to foster educational innovation and digital literacy skills, with the primary goal of improving and transforming the learning processes for the stakeholders. Advanced level of digital technologies and literacy skills for both the educators and learners, respectively, can transform the teaching and learning processes (OECD, 2016). Moreover, one of the main implications and results of this study can be related to the fact that, although the digital technologies offer endless possibilities to facilitate the teaching and learning processes, *their use* requires, also as never before, to look into more pertinent teaching methods and development of teaching-social skills. This means advanced technical knowledge and real-world application of the digital technologies, research, and resources for teaching and learning. Which presumably brings us to the conclusion that the different organizations (e.g. educational institutions, government sectors, policymakers, funding/financial institutions and investors) (IDB, 2019, 2020; LALA, 2020; LASPAU, 2020; Microsoft, 2020; TEC, 2020a) who are either directly or indirectly involved in the design of the educational models and curriculum, promotion, and development of strategies/policies for funding and governing of the several HEIs, particularly in LATAM, must focus their attention towards addressing the aforenoted challenges in order to effectively benefit or advance in use of digital technologies for teaching and learning in practice (Bezanilla et al., 2019; Vilma, 2019).

Theoretically, this study contributes to the *techno-data-structure* or methodological approaches (Raffaghelli et al., 2020) to using information drawn for educational data to inform the higher education research and development. Through the method and results of this paper, higher educational institutions, investors, and policymakers particularly in LATAM, are able to create space and opportunity for *meaning-making* and *theoretical reflection* on the different factors that supposedly influence the use and adoption of digital technologies for teaching and learning in their different contexts (Raffaghelli et al., 2020). Besides, many institutions have experienced transformations in the past as new forms of digital or educational data are being generated, analyzed, and used to support the decision-making processes (Williamson, 2018). Accordingly, the research implications of this study points towards the fact that the educational datasets can be analyzed and/or plays a vital role in understanding/enabling the innovative developments per se, within the clearer context of the teaching and learning processes in LATAM. This includes leveraging the powerful techno-data-structure approaches (Raffaghelli et al., 2020), such as the Text mining method defined in this study.

The two-step (mixed) methodology we introduced in this study opens the way for researchers or educators to embrace a wider and cross-evaluation research method that not only involves the practice of statistically drawing inferences about the studied phenomenon, but also, it integrates an effective Text mining technique that proves useful in analyzing and extraction of relevant information from the educational datasets. Moreover, there are studies, both in theory and in practice, on the idea of analyzing textual datasets to understand patterns/information in respect to the studied phenomenon and decision-making purposes within the several applied contexts or real-world settings (Derakhshan & Beigy, 2019; Fernández-Isabel et al.,; 2019; Lastra-Díaz et al, 2019; Perikos & Hatzilygeroudis, 2016; Tian et al, 2018). In other words, in educational research or scientific studies, that typically focus on analyzing (extracting) information from completed questionnaires with quantitative and qualitative items; it is also very effective to employ the *techno-data-structure* methods (Okoye et al., 2020; Raffaghelli et al., 2020), such as the Text mining technique described in this study (see Section 3.3.1), in understanding/drawing a deeper insight into the users comments or opinions when completing the questionnaires (Fernández-Isabel et al.,; 2019; Pandey & Pandey, 2019; Perikos & Hatzilygeroudis, 2016).

#### Socio-cultural factors and impact

While the findings of this study and the pieces of evidence we drew from the literature (see: Background information) (UNESCO, 2015; IEEE, 2020b; Ertmer, 1999; Mercader & Gairín, 2020, Lakkala & Ilomäki, 2015), purportedly shows that many developed countries, otherwise referred to as high-tech in this study’s context, may have invested in digital technologies especially in education. Considering the socio-cultural perspective, the developing counterparts (low-tech) are still facing a toilsome and portentous task of achieving and benefiting from the vast potentials of the digital technologies in education or learning due to costs of investment and limited infrastructures (Tsegay, 2016). For instance, the implications of such socio-technical or cultural differences could be that in LATAM, higher educational institutions in the developing regions, for instance, Perú are yet to fully profit from the vast benefits of using the digital technologies for teaching and learning (see: Tables 7, 8), although the other countries cannot be fully excluded from the inefficiencies in the use of digital technologies for teaching and learning. In other words, notwithstanding the fact that the different initiatives and strategies for the HEIs and/or educational transformations are promising, countries in low-tech settings are still facing challenges, including limited access to funds and training, limited infrastructures such as internet connectivity and network, and inability of HEIs to utilize the educational resources and datasets to help improve or inform the administrative policies and management in the region or cross-national margins (LALA, 2020).

#### Global educational practice and implications: what is the outlook?

Whereas there is evidence, both in theory and in practice, that digital technologies have become a fundamental and indispensable element of the modern-day education (de Souza Rodrigues et al., 2020; IEEE, 2020b; Munro, 2018; Okoye et al., 2021; UNESCO, 2021a). There still exists the issue of idiosyncratically and pedagogical transformation of the educational ecosystem at large (Boninger et al., 2019, 2020; Cuban, 2020, 2021; Mercader & Gairín, 2020; Molnar & Boninger, 2020; Renz & Hilbig, 2020). HEIs in LATAM are not an exception, as the rigidity of the curriculum (Reisberg, 2019) also presents a threat to ample adoption and implementation of TEL for education in the region (LALA, 2020). Nevertheless, we must acknowledge the fact that TEL have allowed the educators to continue business, and delivery of academic services remotely, in addition, to ensuring that the teachers and students stay safe and healthy whilst learning, particularly during the recent pandemic (Chick et al., 2020; IEEE, 2020b; Reimers et al., 2020; Setiawan, 2020; UNESCO, 2020, 2021b). Thus, any effort by HEIs to implement and sustain a continuous quality education must embrace and include the innovations, or yet, TEL-based initiatives, that have spanned during this time.

As an example, UNESCO in its Global Education Coalition (GEC) initiative (UNESCO, 2021a) stated that never before have the educational system witnessed disruption at this (large) scale, and partnership amongst the concerned stakeholders is the only way forward. In this vein, they (UNESCO) called for “coordinated” and “innovative” actions that are aimed to unlock solutions that are not only used to support teachers and students with the teaching-learning processes, but through the recovery process, and in the longer term, with a principle focus on inclusion and equity (UNESCO, 2021a). Indeed, those actionable efforts per se, must include ensuring that the stakeholders (teachers, students, educational community), particularly those in low-tech areas or regions, have access to adequate digital literacy, technologies, and network infrastructures (IEEE, 2020b; LALA, 2020; Sánchez-Cruz et al., 2021) as uncovered in this study. Apparently, this also includes ensuring effective delivery of online instructions, and management of unforeseen challenges that may inadvertently emerge in the different TEL-based platforms (Bao, 2020; Engen, 2019; Pettersson, 2020; Toit & Verhoef, 2018). Moreover, the didactical recovery and/or lessons learned due to the rapid shift from traditional classrooms to remote learning during the recent pandemic (Shambour & Abu-Hashem, 2022), have revealed the need for educators to not only ensure that the institutions do not replicate the unreadiness and/or inability to effectively implement TEL, but rather hand-in-hand, should aim to leverage the resultant technologies and innovations to build towards an improved educational ecosystem that is capable of nurturing the stakeholders (teachers, students, the education community, society, etc.) into global citizens and/or life-long learners (OECD, 2020b, 2021; Rogers & Shwetlena, 2020; UN, 2021; UNESCO, 2014, 2016, 2020; UNICEF, 2018).

In other words, whilst the results of this study and pieces of evidence we drew from the literature (see: Background information, Results and Discussion - Sections 2.3 and 4), shows that the transition and/or effective use of TEL to foster delivery of the educational programs will heighten the teaching and learning processes across the different HEIs. On the other hand, there is now, never as before, the need for HEIs and policy makers to innovate, develop, and implement adequate solutions towards addressing the problems (reach, barriers, and bottleneck) with use of digital technologies in Education as identified in this study. In the wider spectrum, The Organization for Economic Cooperation and Development (OECD) have recently highlighted in its policy responses to the recent global pandemic, the long-term effects the crisis could have on the future of Science, Technology, and Innovation (STI) to include; challenges that has to do with accelerating digital transformation, literacy and skills e.g. in education, increased importance of technology in the economy and society, and prevalence of inequalities in access to and the use of digital technology (OECD, 2021).

### Limitations and suggestions for future research

The study acknowledges some of the limitations that may come with the work done in this study. First, based on the research design and scope of this study; the authors conclusions and recommendations are based only on the collected data sample from the survey questionnaire we administered in the LATAM context, and based on the research questions and hypotheses. Moreover, considering the aforenoted study’ context and aim for which the survey questionnaires and items were designed, the analysis of the survey items may appear to point to a number of opportunities for refining the instrument for use in future researches or regional contexts. Henceforth, in future studies, for instance, the authors could consider extending the survey items or questionnaire, and applying the research survey to cover other parts of the world in order to understand the current situation in the different global and/or diverse contexts. This is due to the fact that there could be not or no much differences in the other countries or regions around the world, compared to LATAM. Moreover, ascertaining a conceptual knowledge of the existing status quo and digital initiatives from the other countries and regions apart from LATAM, could provide us with more data and details to identify some possible differences or factors that may have not already been identified in this study.

Second, although the study has applied a non-parametric test to analyze the collected data due to its distribution-free, and ability to produce statistically significant results without being affected by outliers. When taking into account the variations and countries in which the analyzed sample size were considered small compared to others. We note that notwithstanding the sample sizes, all the studied countries verge to add an idea of what the educators consider to be the impact of digital technologies in their respective education system and countries, which we believe to be very important to the objective of our study, and also allows us to consider new areas of opportunity for future research.

Finally, it is noteworthy to mention that following the inevitable digital necessities by the stakeholders, educators, and investors, particularly at the recent and unprecedented time of the pandemic or post-pandemic education. We have implemented in another research (Okoye et al, 2021), by investigating how the different contingency strategies and rapid shift from the traditional model or face-to-face education, to technology-mediated education have changed the users’ perspective, learning progression, and experiences in teaching and learning, especially in LATAM, and potentially in the future, in other parts of the world.

## Conclusion

This study employed a two-step methodology that was grounded on the descriptive and diagnostic research approach (Narayan, 2019) within the positivism framework (Elden, 2009; Outhwaite, 2015; Turner, 2001), to evaluate the opinions and perspectives of the educators towards existing challenges and advances in use of digital technologies for teaching and learning across HEIs in LATAM. Practically, we explored the differences in challenges that users face across the various studied countries, by considering the extent of reach or demographics, potential barriers, and bottlenecks. The results of the study’ analysis show that factors such as limited training and resources, access to internet and infrastructures contributed significantly to the challenges or level of adoption of digital technologies for education across HEIs in the LATAM region. Thus, we uncovered the potential barriers and bottlenecks on why TEL-based education may not be effectively implemented in the HEIs, and then empirically discussed the different actionable strategies or methods that educators can adopt to meet those challenges. The study also shows the benefit of data-structure approach such as the Text mining technique, and its application within the education domain to understand the impact of digital technologies on the teaching and learning processes. The outcome of this study is relevant to support the different operational policies, regulations, research, and decision-making strategies, for both the educators, financial investors, and policymakers, to uphold TEL-based education, Educational Technologies, and Teaching/learning Process Innovations.

## Data Availability

The datasets used and analyzed during this study are available from the corresponding author on request.

## Appendix 1

**Research Instrument (Survey Questionnaire)**

## Appendix 2

**Most frequent terms used to describe use of digital technologies for teaching-learning in LATAM broken down by Country.**

## Appendix 3

**Classification of Emotions expressed by participants towards the use digital technologies for teaching and learning in LATAM by Country.**

## Appendix 4

**Dunn (1964) - Kruskal-Wallis multiple comparison, p-values adjusted with “Bonferroni” method, Q3 = Country**

**Q18 = dunnTest (Q18 ~ Q3, data = BIDStudy, method = "bonferroni")**

**Q20 = dunnTest (Q20 ~ Q3, data = BIDStudy, method = "bonferroni")**

**Q21 = dunnTest (Q21 ~ Q3, data = BIDStudy, method = "bonferroni")**

**Q27 = dunnTest (Q27 ~ Q3, data = BIDStudy, method = "bonferroni")**

**Q28 = dunnTest (Q28 ~ Q3, data = BIDStudy, method = "bonferroni")**

**Q30 = dunnTest (Q30 ~ Q3, data = BIDStudy, method = "bonferroni")**

**Q32 = dunnTest (Q32 ~ Q3, data = BIDStudy, method = "bonferroni")**

**Q33 = dunnTest (Q33 ~ Q3, data = BIDStudy, method = "bonferroni")**

## Code availability

The code used in performing the text mining analysis in this study are available from the corresponding author on request.

## Declarations

### Originality and review status

The authors confirm that this work is original and has not been published elsewhere, nor is it currently under consideration for publication elsewhere.
